# Indications and recent evidence for apheresis in children and adults with kidney diseases: a comprehensive review

**DOI:** 10.1093/ckj/sfaf282

**Published:** 2025-09-10

**Authors:** Rupesh Raina, Eugene Yu-hin Chan, Jieji Hu, Pujan Moradiya, Bryce Pember, Priyanka Khandelwal, Ruchi Mahajan, Ali Düzova, Kinnari Vala, Vivekanand Jha, Olivia Boyer, Sidharth Sethi, Pietro Canetta

**Affiliations:** Department of Nephrology, Akron Nephrology Associates at Cleveland Clinic Akron General Medical Center, Akron, OH, USA; Department of Pediatric Nephrology, Akron Children’s Hospital, Akron, OH, USA; Department of Paediatrics, Faculty of Medicine, The Chinese University of Hong Kong, Shatin, Hong Kong SAR; Paediatric Nephrology Centre, Hong Kong Children’s Hospital, Kowloon Bay, Hong Kong SAR; Department of Nephrology, Akron Nephrology Associates at Cleveland Clinic Akron General Medical Center, Akron, OH, USA; College of Medicine, Northeast Ohio Medical University, Rootstown, OH, USA; College of Medicine, Northeast Ohio Medical University, Rootstown, OH, USA; College of Medicine, Northeast Ohio Medical University, Rootstown, OH, USA; Division of Nephrology, Department of Pediatrics, The Hospital for Sick Children, Toronto, Canada; Department of Pediatric Nephrology, Atrium Health, Levine Children’s Hospital, Charlotte, NC, USA; Division of Pediatric Nephrology, Faculty of Medicine, Hacettepe University, Ankara, Türkiye; Department of Pediatric Nephrology, Institute of Kidney Diseases and Research Centre & Gujarat University of Transplantation Sciences, Ahmedabad, India; George Institute for Global Health, UNSW, New Delhi, India; School of Public Health, Imperial College, London, UK; Prasanna School of Public Health, Manipal Academy of Higher Education, Manipal, India; Néphrologie Pédiatrique, Centre de Référence du Syndrome Néphrotique Idiopathique de L’enfant Et L’adulte, Hôpital Necker - Enfants Malades, APHP, Inserm U1163, Institut Imagine, Université Paris Cité, Paris, France; Division of Pediatric Nephrology, Kidney Institute, Medanta, the Medicity Hospital, Gurgaon, Haryana, India; Department of Medicine, Division of Nephrology, Columbia University Irving Medical Center, New York, NY, USA

**Keywords:** apheresis, LDL-apheresis, plasmapheresis, therapeutic plasma exchange

## Abstract

**Introduction:**

Plasmapheresis has been a therapeutic option in kidney diseases to eliminate disease-causing autoantibodies, circulating factors, and abnormal components involved in complement pathways. We aim to systematically review the effectiveness and adverse events associated with plasmapheresis and related apheresis therapies in treating kidney diseases in paediatric and adult populations.

**Methods:**

We searched databases including EMBASE, CINAHL, PubMed, and Cochrane Central for studies from 2010 to October 2023. The search terms included terms related to glomerulonephritis treated with plasmapheresis. Outcomes included the patient’s length of hospital stay, mortality, development of kidney failure, associated comorbidities, and adverse events. Risk of bias was assessed using the Newcastle–Ottawa Scale, and meta-analyses were performed to calculate pooled adverse event rates.

**Results:**

A total of 33 studies with 1363 participants were included. The pooled proportion of kidney failure was 26.36% (95% CI 17.38%–36.47%), and the rate of dialysis requirement was 30.43% (95% CI 14.80%–48.82%). The mortality rate was 10.86% (95% CI 9.12%–12.81%). Adverse events were reported in 31.03% (95% CI 12.78%–53.05%) of cases. Heterogeneity was significant for most outcomes. We also performed a literature review due to a lack of adequate studies regarding the use of plasmapheresis in lupus nephritis, multiple myeloma, and atypical haemolytic uremic syndrome, as well as the use of low-density lipoprotein apheresis in kidney diseases.

**Discussion:**

Plasmapheresis has demonstrated remission in patients with kidney diseases, particularly in those with ANCA-associated vasculitis and FSGS. Based on the results of our systematic review, we discuss the use of plasmapheresis for treating glomerular diseases, atypical haemolytic uremic syndrome, other kidney diseases, and the usage of low-density lipoprotein apheresis. Further research is needed to improve patient outcomes and reduce complications, especially in paediatric populations.

## INTRODUCTION

Apheresis is a broad term describing the extracorporeal removal of a specific component from the blood [1]. Plasmapheresis refers specifically to the removal of plasma from the patient. Therapeutic plasma exchange, on the other hand, involves removing the patient’s plasma and replacing it with a substitute fluid. Plasmapheresis has become an integral therapeutic option in various kidney diseases to remove disease-causing autoantibodies, circulating factors, and abnormal components along the complement pathways. Plasma can be separated from blood using either a centrifugation system, commonly used in the USA, or membrane separation, more prevalent in Japan and Europe [2]. Plasmapheresis also exerts immunomodulatory effects, as it has been associated with various autoimmune diseases, showing a decline in B cells and NK cells, an increase in T cells, enhanced T suppressor cell function, and a rise in regulatory T cells [3]. The current American Society for Apheresis guidelines and Kidney Disease: Improving Global Outcomes (KDIGO) guidelines provide comprehensive frameworks for the application of apheresis in clinical practice, with other societies such as the International Pediatric Transplant Association providing additional apheresis considerations in kidney conditions such as recurrent focal segmental glomerulosclerosis [4–6]. Given these therapeutic possibilities, a comprehensive evaluation of plasmapheresis in kidney diseases is crucial to better understand its role in managing both immune-mediated and non-immune kidney conditions. Therefore, our objective is to systematically review the efficacy, safety, and clinical outcomes of plasmapheresis in treating both paediatric and adult patients with focal segmental glomerulosclerosis (FSGS), ANCA-associated vasculitis (AAV), rapidly progressive glomerular nephritis (RPGN), post-kidney transplant, and anti-glomerular basement membrane disease (anti-GBM) focusing on treatment outcomes, kidney failure, and adverse events. In addition, we discuss the use of plasmapheresis to impact the treatment of recurrent FSGS, diffuse alveolar haemorrhage, ABO-incompatible kidney transplants, and atypical haemolytic uremic syndrome (aHUS) via different techniques.

### Methods

#### Included studies

Following Preferred Reporting Items for Systematic Reviews and Meta-Analysis (PRISMA) guidelines, we conducted a systematic review using databases including PubMed/MEDLINE, Web of Science, Cochrane Library, and CINAHL. Our search focused on various forms of glomerulonephritis and related renal replacement therapies, including plasmapheresis, across studies published in English. We followed the PRISMA guidelines (Supplemental Files).

#### Formation of discussion

After conducting our systematic review of the literature, we summarized the limited studies and patients on the use of plasmapheresis in paediatric and adult glomerulonephritis in Table 1. Many of the studies included are case reports and case series, which limits the generalizability of the findings. Therefore, the focus of our discussion is to provide clinicians with actionable guidance, while acknowledging the heterogeneity of the available data, given the modest benefit of plasmapheresis in terms of remission. Moreover, remission rates reported across studies varied, and differing definitions of complete and partial remission were used.

**Table 1:** Details about different parameters in studies across different indications.

Indication	Number of studies	Sample size	Mean/median age (years)	Patients who required dialysis	Follow-up period (months)
ANCA-associated vasculitis	10	821	12–72	42.8% [142/332] across 5 studies	12–36
Renal transplant	9	297	9–52	–	21–48
Anti-GBM	6	152	29–52	68.4% [52/76] across 5 studies	12–41
FSGS	6	63			24–36
RPGN and crescentic IgA nephropathy	5	91	40–51	55.1% [27/49] across 2 studies	16–36

The 2021 KDIGO guidelines emphasize the selective role of plasmapheresis in glomerular diseases, with strong support for its use in anti-GBM disease (Grade 1C recommendation) [7]. By contrast, evidence supporting plasmapheresis in treating other glomerular diseases such as IgA nephropathy, IgA vasculitis nephritis, lupus nephritis, and steroid-resistant nephrotic syndrome, is less definitive [7–10]. Our discussion builds on these guidelines by addressing the practicalities of applying plasmapheresis in real-world clinical settings, particularly for dialysis-dependent patients, and incorporating additional therapies such as rituximab and immunoglobulin to mitigate autoantibody production in the appropriate disease states. For example, in the paediatric population, both centrifugation and membrane filtration techniques are used, although centrifugation is preferred in smaller patients due to lower extracorporeal volume and blood flow requirements [11–13]. On the basis of the child’s size, careful selection of vascular access and consideration of extracorporeal circuit volume is needed to avoid hemodynamic instability. Supplemental Table S11 outlines additional case reports and case series on the use of plasmapheresis in paediatric glomerular diseases. We call for further studies to refine treatment strategies for paediatric glomerular diseases and offer detailed clinical insights into conditions in which plasmapheresis and other apheresis techniques, such as LDL-apheresis, may provide further benefits.

Finally, we discuss lupus nephritis, multiple myeloma, and atypical haemolytic uremic syndrome, conditions where plasmapheresis and other apheresis techniques including LDL-A may provide a benefit. We aim to equip clinicians with a clearer, more nuanced approach to using plasmapheresis, ensuring individualized treatment while acknowledging the current limitations in the literature as found in our systematic review.

### Summary of results

A total of 33 studies (12 conducted before 2015, 13 during 2015–2019 and 8 studies post 2019) were included. The total sample size of all the included studies was 1363 ranging from 3 to 352 across the studies. The mean/median age of the included subjects ranged from 9 to 72 years and 59.1% of the subjects were males. The pooled proportion (95% CI) of remission was 60.65% (47.35%–73.18%) [*I*^2^: 85.77% (78.66%–90.51%); *P* < .0001; 17 studies; *N* = 420; Supplemental Table S4; Supplemental Fig. S1a]. The pooled proportion (95% CI) of kidney failure was 26.36% (17.38%–36.47%) [*I*^2^: 89.99% (85.71%–92.98%); *P* < .0001; 18 studies, *N* = 1005; Supplemental Table S5; Supplemental Fig. S2a] across all the included studies. The pooled proportion (95% CI) of dialysis requirement was 30.43% (14.80%–48.82%) [*I*^2^: 91.24% (87.06%–94.07%); *P* < .001; 14 studies; *N* = 356; Supplemental Table S6; Supplemental Fig. S3a]. The pooled proportion (95% CI) of mortality was 10.86% (9.12%–12.81%) [*I*^2^: 48.42% (19.31%–67.03%); *P* = .0028; 27 studies; *N* = 1114; Supplemental Table S7; Supplemental Fig. S4a]. The pooled proportion (95% CI) of adverse events was 31.03% (12.78%–53.05%) [*I*^2^: 92.93% (88.74%–95.56%); *P* < .0001; nine studies; *N* = 344; Supplemental Table S8; Supplemental Fig. S5a]. LDL-A was not included in the systematic review; instead, a summary point was generated based on a targeted review of available evidence and informal expert input. Additional results can be found in the Supplemental Files.

## DISCUSSION: THE USAGE OF PLASMAPHERESIS FOR KIDNEY DISEASE

### Antineutrophil cytoplasmic antibody-associated vasculitis

There were 10 studies with a total of 821 patients ranging from the age of 12 to 72 years with AAV who were treated with plasmapheresis. Patients with other forms of RPGN were excluded. Patients often present with rapidly progressive glomerulonephritis due to pauci-immune crescentic glomerulonephritis [14]. Approximately 40% of patients with RPGN are diagnosed with pauci-immune RPGN, typically classified as granulomatosis with polyangiitis, microscopic polyangiitis, or eosinophilic granulomatosis with polyangiitis, and these patients generally have a poor prognosis [15]. The two major antigens targeted by ANCA are proteinase 3 (PR3) and myeloperoxidase (MPO), which are expressed in neutrophil primary granules and monocytes.

The MEPEX trial, which included 137 patients with serum creatinine >500 μmol/l (5.8 mg/dl), established the beneficial role of plasma exchange (PLEX) in severe kidney vasculitis, showing that 69% of patients randomized to PLEX had kidney recovery (defined as patient survival, dialysis independence, and serum creatinine <5.8 mg/dl) at 3 months compared to 49% of those randomized to intravenous methylprednisolone (95% CI for the difference 18%–35%; *P* = .02), with a 24% risk reduction for progression to kidney failure at 1 year (95% CI 6.1%–41%) [16]. PLEX has been shown to significantly reduce the risk of end‐stage kidney disease at 3 months (two studies: RR 0.43, 95% CI 0.23–0.78) and 12 months (six studies: RR 0.45, 95% CI 0.29–0.72) [17].

A landmark randomized controlled trial, PEXIVAS, examined different corticosteroid dosing regimens and the role of plasmapheresis in adults with severe AAV involving the kidneys or lungs [18]. Results from the trial did not demonstrate a reduction in the incidence of death or kidney failure after plasmapheresis (hazard ratio 0.86; 95% CI 0.65–1.13).

Walsh *et al.* subsequently performed a meta-analysis and systemic review that included 1060 subjects enrolled in nine clinical trials [19]. While plasmapheresis did not influence mortality, the risk of reaching kidney failure at 12-months was significantly reduced (RR 0.62; 95% CI 0.39–0.98). However, there was an increased risk of serious infection by 27%. This is supported by our systematic review sensitivity analysis finding a pooled proportion of adverse events of 68.8% (54.7%–81.4%) [*I*^2^: 75.6% (0.00%–94.5%); *P* = .043; two studies; *n* = 216]. In children, treatment of AAV is heterogeneous with 34% of children treated with plasmapheresis as induction in an international study evaluating 337 children from 41 countries [14].

It appears that plasmapheresis is reserved for severe cases of AAV, with an analysis of 975 adult cases showing patients treated with plasmapheresis were more likely to have experienced acute kidney injury (AKI) (96% vs. 90%, *P* = .0007), AKI requiring dialysis (52% vs. 16%, *P* < .001), hypoxia (40% vs. 16%, *P* < .0001), and respiratory failure requiring mechanical ventilation (13% vs. 3%, *P* = .0003) [20]. Sensitivity analysis of kidney failure showed a rate of 19.6% (16.7%–22.9%) [*I*^2^: 45.4% (0.0%–77.0%); *P* = .0885; seven studies; *n* = 646]. Sensitivity analysis of dialysis requirement showed a rate of 24.0% (10.1%–41.5%) [*I*^2^: 83.6% (62.8%–92.8%); *P* = .0001; five studies; *n* = 231]. Sensitivity analysis of mortality showed 10.6% (8.6%–12.9%) [*I*^2^: 21.5% (0.0%–61.5%); *P* = .245; 10 studies; *n* = 821]. Unmet needs in this field include a need for more data in the paediatric population, as well as standardization of plasmapheresis dosage and duration.

### Anti-glomerular basement membrane disease

There were six studies with a total of 152 patients ranging from the ages of 29 to 52 with anti-GBM disease, previously known as Goodpasture syndrome, who were treated with plasmapheresis. Patients with other forms of RPGN were excluded. Anti-GBM disease is an autoimmune condition where antibodies target the glomerular basement membrane (type IV collagen). This results in pulmonary–renal syndrome, where there is often a clinical presentation of rapidly progressive glomerulonephritis and/or pulmonary haemorrhage.

Earlier studies of treating anti-GBM disease with plasmapheresis reported favourable outcomes, whereas subsequent studies showed conflicting findings, especially among patients with significant global glomerulosclerosis (>50%) who often experienced poor kidney outcomes. In a retrospective study of 972 patients, PLEX was the most common modality of apheresis (89%, *n* = 863), while fewer received IA (4%, *n* = 37) or a combination of both (7%, *n* = 72), with IA used in 28.9% of centres [21]. A significant portion of patients, 13.9%, had double positivity for granulomatosis with polyangiitis or microscopic polyangiitis, 45.0% required ICU care, 77.8% underwent kidney replacement therapy (KRT), 71.1% needed red blood cell transfusions, 26.9% required mechanical ventilation, 40.4% had long-term KRT access, and in-hospital mortality was 10.4% [21].

In a retrospective study of 207 patients, patients who underwent PLEX had lower in-hospital mortality than those who did not with a risk difference of 17.5% (95% CI 2.6%–32.4%; *P* = .022) [22]. PLEX and IA were associated with similar rates of ICU treatment (44.1% for PLEX vs. 43.2% for IA), mechanical ventilation (26.8% for PLEX vs. 18.9% for IA), and in-hospital mortality (10.4% for PLEX vs. 10.8% for IA). PLEX was significantly linked to more extrapulmonary bleeding events (OR 3.08, 95% CI 1.36–8.28, *P* = .01), greater need for RBC transfusion (OR 2.48, 95% CI 1.26–4.91, *P* = .008), and higher in-hospital RRT use (OR 2.47, 95% CI 1.17–5.11, *P* = .02) [21]. In a retrospective study of 28 patients, double filtration plasmapheresis (DFPP) had similar efficacy of clearing anti-GBM antibody (59.0% vs. 71.2%, *P* = 1.00) compared to IA, with fewer patients in the DFPP group experienced reduced IgG (62.7% vs. 83.5%, *P* = .002) [23].

Globally, the usage and timing of other immunosuppressive regiments such as prednisone, rituximab, and cyclophosphamide differs [7, 24]. In a retrospective study with 221 patients, the combination therapy had an overall beneficial effect on both patient survival [hazard ratio (HR) for patient mortality, 0.31; *P* = .001] and kidney survival (HR for kidney failure, 0.60; *P* = .032), particularly patient survival for those with Goodpasture syndrome (HR for patient mortality, 0.29; *P* = 0004) and kidney survival for those with anti-GBM nephritis with initial serum creatinine >6.8 mg/dl (HR for kidney failure, 0.52; *P* = .014) [25]. This suggests the benefits of combination therapy, and early identification of IgG anti-GBM autoantibodies and other pathogenic anti-GBM Ig subtypes (IgA, IgM, and alpha 4 or 5 NC1) [26].

Plasmapheresis can also be used in patients with both AAV and anti-GBM antibodies. The epidemiology varies, with up to 5% of ANCA-positive patients also having anti-GBM antibodies, and 32% of anti-GBM-positive patients also having detectable ANCA antibodies [27]. Uto *et al.* performed a literature search and found 52 double-positive patients with MPO-ANCA antibodies, with a survival rate of 74% (26 out of 35) for patients treated with both immunosuppressive drugs and PLEX, as opposed to a survival rate of 53% (9 out of 17) for those who received only immunosuppressive drugs [28]. KDIGO guidelines state that patients with both AAV and anti-GBM antibodies should be treated aggressively with PLEX and immunosuppression, with greater chance of kidney recovery compared to patients with anti-GBM antibodies alone but a higher relapse rate [7]. There is a need for more data in the paediatric population, as well as standardization of plasmapheresis dosage and duration.

### Rapidly progressive glomerulonephritis

In our systematic review, there was a total of five studies with a total of 91 patients ranging from the ages of 40 to 51 with RPGN. Most patients with rapidly progressive immunocomplex glomerulonephritis present with clinical or pathological evidence of a primary glomerulonephritis or systemic immune complex disease, such as systemic lupus erythematosus [29]. In particular, plasmapheresis has been noted to be useful in the crescentic course of conditions like lupus, cryoglobulinemia, and IgA nephritis/Henoch–Schoenlein purpura.

In a meta-analysis of 387 patients, the relative risk for kidney failure or death was 0.80 for patients with idiopathic RPGN (studies included had >75% of patients with RPGN that were reported pauci-immune or idiopathic) treated with adjunctive PLEX compared to standard care (95% CI, 0.65–0.99; *P* = .04), with no significant heterogeneity detected (*P* = .5; *I*² = 0%) [30]. The RR for kidney failure alone was 0.64 (95% CI, 0.47–0.88; *P* = .006), while the RR for death alone was 1.01 (95% CI, 0.71–1.4; *P* = .9). In a systematic review of 64 patients with rapidly progressive IgA nephritis, 42.1% (*n* = 27/64) achieved remission, with 20.3% (*n* = 13/64) achieving complete remission (in general, proteinuria  0.3 g/day) and 18.7% (*n* = 12/64) partial remission (in general, proteinuria 0.3–0.5 g/day and 50% reduction from baseline), while 60.9% (*n* = 39/64) progressed to kidney failure [31]. There is a need for more data in the paediatric population, as well as standardization of plasmapheresis dosage and duration.

### Kidney transplant

In our systematic review, there was a total of nine studies with 297 patients ranging from the ages of 9 to 52 who used plasmapheresis before or after kidney transplant. Plasmapheresis has been used to treat antibody-mediated rejection (AMR) and, as part of desensitization protocols for ABO-incompatible kidney transplants and patients with preformed anti-HLA antibodies [32]. Although evidence for the benefit of plasmapheresis is growing, the indications remain unclear. In AMR, several trials suggest plasmapheresis, usually combined with intravenous immunoglobulin, can reverse rejection in 55%–100% of cases [33]. In a retrospective study of 49 patients, Kakkar *et al.* found that 6 months post-transplant, the patient and graft survival were 93.3% and 89.5%, whereas at 12 months, they were 89.3% and 81.5%, respectively, in patients with biopsy-proven acute humoral rejection [34]. A prospective study by Bennani *et al.* found that donor specific antibodies were decreased [mean fluorescence intensity declined from 9160 (4000–15 400) at baseline toto 7375 (215–18 100) (*P* = .018) at day 45, and to 4060 (400–7850) (*P* = .001) at 1 year], but there was no difference in kidney function at 1 year after treatment with DFPP [35]. However, there is a paucity of high quality randomized control trials for the use of plasmapheresis in AMR [36].

Plasmapheresis has also enabled transplantation in highly sensitized patients with preformed anti-HLA antibodies, often combined with immunosuppressive drugs. This approach has led to 1-year graft survival rates exceeding 70% [33]. Similarly, plasmapheresis is used to deplete anti-A or anti-B antibodies in ABO-incompatible transplants, with graft survival around 80% at one year. Depending on the baseline anti-A/B antibody titres, the target for an antibody level is ≤1:8 on the day of transplant [3]. In a retrospective study of 142 patients with high-panel reactive antibodies, the HR for graft rejection was 2.37 (95% CI 0.89 to 6.35) and 2.22 (95% CI 0.54 to 9.13) [37].

In addition, plasmapheresis appears to have long-term benefits post-kidney transplant. Patients treated with additional anti-CD20 therapy and plasmapheresis compared to standard immunosuppression with IVIg had lower microcirculation inflammation lesions (glomerulitis and capillaritis score of 1.8 ± 0.2 vs. 2.7 ± 0.2, respectively, *P* = .03), a lower rate of transplant glomerulopathy (7% vs. 38%, *P* = .02), and a lower rate of chronic AMR (41.3% vs. 13.3%, respectively, *P* = .03) [38]. In acute rejection, antibody removal with plasmapheresis has been shown to improve graft survival over a long-term period (*n* = 92; HR 0.46; 95% CI 0.26–0.82; *P* = .009) [39].

### Focal segmental glomerulosclerosis

There were six studies with a total of 63 patients with FSGS in our systematic review. In those studies, most patients received plasmapheresis after kidney transplant, with few patients receiving plasmapheresis in the native kidney. In two studies, patients received LDL-apheresis, including post-transplant patients. Recurrence of FSGS may occur within hours after kidney transplant in up to 30%–60% of patients and is a sinister condition associated with graft loss [40]. In primary FSGS, it is hypothesized that circulating permeability factors cause progressive podocyte injury [41]. Anti-nephrin autoantibodies have been identified as a potential circulating factor and play a role in post-transplant recurrence of FSGS. Other potential candidate factors include serum soluble urokinase receptor, vasodilator-stimulated phosphoprotein, apolipoprotein A-I, galactose, and cardiotrophin-like cytokine-1 [42]. Laboratory assays to identify these permeability factors are unavailable clinically. A systematic review of 85 patients showed that a combination of rituximab and plasmapheresis in patients with post-transplant FSGS who were already on immunosuppressant such as calcineurin inhibitors resulted in a similar overall remission rate of 72.7% (95% CI 52.3%–86.6%), including 41.0% complete remission (proteinuria <1 g/day) and 31.7% partial remission (reduction in proteinuria >50% from peak, but >1 g/day), along with significant reductions in serum creatinine levels. The mean difference in serum creatinine pre- and post-treatment was −0.65 mg/dl (95% CI −1.15 to −0.14), while the mean reduction in proteinuria was −4.79 g/day (95% CI −7.02 to −2.56) [43].

However, in our analysis, pre-transplant plasmapheresis does not show a significant effect in preventing recurrent FSGS post-transplant. A systematic review of 571 mostly adult patients, most without genetic testing, found that plasmapheresis alone was not associated with any significant difference in FSGS recurrence when compared with no plasmapheresis with pooled risk ratio was 0.85 (95% CI 0.60–1.21, *I*^2^ = 23%) [44]. There is an ongoing clinical trial to assess the use of plasmapheresis with rituximab for preventing FSGS recurrence [45].

In another systematic review of 423 patients treated with PLEX, 198 (46.8%) achieved complete remission (proteinuria <0.5 g/day) and 119 (28.1%) achieved partial remission (proteinuria <3.5 g/day), resulting in an overall remission rate of 71.0% (95% CI 66.2%–75.4%) [46]. In a restricted sample of 235 patients, remission rates were similar between 116 adults (69.1%, 95% CI 59.6%–77.2%) and 119 children (70.2%, 95% CI 61.1%–77.9%), with genetic data not available [46]. The total number of PLEX sessions ranged from 1 to 200 with a median of 12 sessions (IQR 10, 20), showing a wide range of treatment duration. Remission was calculated using a random effects model.

A retrospective study of 176 patients aged ≥16 years with FSGS treated with plasmapheresis with or without rituximab did not show an association between early biopsy-proven FSGS recurrence (median time to recurrence 1.5 months) and response to treatment (odds ratio [OR], 0.83; 95% CI, 0.24 to 2.85; *P* = .77) [40]. However, this study suggests that non-standardized treatment approaches and differences in treatment intensity and length may affect outcomes in recurrent FSGS.

Some patients who are treated with plasmapheresis cannot be weaned off, as proteinuria recurs shortly after cessation of treatment [47]. Chronic plasma-apheresis may be a viable option. In a study of 27 patients with recurrent FSGS who were treated with long-term plasmapheresis, 39% remained on active treatment and 43% could be successfully weaned, whereas 13% experienced therapy failure (defined as increasing proteinuria levels; median duration 23 months; starting frequency of apheresis three sessions per week, with variable tapers; median plasmapheresis frequency three sessions per month) [48]. Notably, some patients could be weaned off plasmapheresis after a slower taper and still maintained disease remission. Chronic plasmapheresis may be an option for patients with recurrent FSGS, and a slow taper may be indicated to achieve remission; however, more research is necessary. We discuss the use of other apheresis modalities such as LDL-A to treat FSGS in a subsequent section.

## OTHER APPLICATIONS OF PLASMAPHERESIS

Plasmapheresis may also be used in various other conditions. Although the available studies on these conditions are limited, precluding a systematic review, we believe it is important to highlight their potential relevance.

### Lupus nephritis

Current KDIGO 2024 guidelines do not recommend the use of plasmapheresis in lupus nephritis [5]. Clinical trials have failed to show any benefit when compared to standard therapy of prednisone and cyclophosphamide [49]. However, there may be a significant subset (up to 20%) of patients with severe lupus nephritis who fail to respond to the conventional therapy, who may have concurrent APS nephropathy and could potentially benefit from adjunctive TPE [50]. In addition, in rare lupus manifestations such as thrombotic thrombocytopenic purpura, plasmapheresis may be used to replace defective ADAMTS13 and abnormal VWF multimers with normal ADAMTS13. A randomized controlled trial by Rock *et al.* resulted in a higher 6-month survival rate compared to plasma infusion, 78.4% versus 62.7% [51]. In a study of 70 patients, survival analysis showed plasmapheresis was a beneficial factor (HR 12.9, 95% CI 2.4–69.8, *P* =  .003) [52]. Plasmapheresis was further shown to be an independently beneficial factor associated with long-term outcomes (HR 8.914, 95% CI 3.028–26.247, *P* <  .001).

### Multiple myeloma

The main cause of AKI in patients with multiple myeloma is the overproduction of nephrotoxic monoclonal light chains, which precipitate in kidney tubules. Kidney recovery occurs in 25%–58% of patients with supportive measures and antimyeloma therapy, particularly when early reductions in serum monoclonal light chains are achieved [53]. Treatments like plasmapheresis and high cut-off dialysis filters, combined with chemotherapy regimens including anti-plasma cell agents such as bortezomib, promote recovery based on the degree of free light chain reduction [54]. In a retrospective study by Premuzic *et al.*, patients receiving plasmapheresis with chemotherapy showed significant eGFR improvements, with a significantly decreased free light chain level in the plasmapheresis and chemotherapy group (34 mg/dl vs 182 mg/dl, *P* < .001) [55]. There was a significantly lower risk of dialysis requirement in patients treated with plasmapheresis (risk ratio = 0.45; 95% CI 0.23–0.86, *P* = .02) [56]. There is a need for further research in the use of plasmapheresis in multiple myeloma and in monoclonal gammopathy of renal significance, especially after the development of modern pharmacologic treatment regimens that rapidly reduce light chain burden.

### Atypical haemolytic uremic syndrome

Atypical haemolytic uremic syndrome (aHUS), a form of thrombotic microangiopathy (TMA) that often leads to AKI, includes a variant associated with anti-factor H antibodies. Anti-factor H antibodies bind to the C terminus of FH, impairing the interaction of factor H with C3b and causing dysregulation and overactivity of the complement pathway [57]. In 44 patients, the mean anti-factor H antibody titre at disease onset was 5000 AU/ml (range 2121–163 829 AU/ml), with follow-up titres at 3 months (*n* = 42), 6 months (*n* = 47), and 12 months (*n* = 23) being 409 (254–861), 277 (154–893.6), and 408 (262–691.8) AU/ml respectively, and the average time from onset to haematological remission (platelet count >100 000/mm^3^, schistocytes <2%, and LDH less than upper limit of normal on two consecutive days) was 27 (17–41) days [58]. In a retrospective study of 45 patients following a median of nine PLEX sessions, anti-CFH antibody titres declined by 87.1% to 414.6 (IQR 251.6–1368.2) AU/ml (*P* < .0001) [59]. Khandelwal *et al.* retrospectively found that younger children were not at increased risk of adverse events from PLEX treatment [60]. Time to PLEX ≥14 days (HR 2.09; *P* = .071) and PLEX for <14 days (HR 2.60; *P* = .017) were predictors of adverse kidney outcomes, while combined PLEX and immunosuppression improved long-term outcomes (HR 0.37; *P* = .026), and maintenance therapy significantly reduced the risk of relapses (HR 0.11; *P* < .001) [58]. This suggests early initiation of PLEX with an adequate duration of therapy leads to improved outcomes. However, Johnson *et al.* in an audit found that of the 12 children with the poorest kidney outcomes (dialysis-dependent by day 33), only three received early, high-volume PLEX in the first 5 days, and two received no plasma therapy at all [61]. Furthermore, anti-factor H antibodies are present in aHUS in only ∼20% of patients, with 50% of the aHUS cases arising due to genetic mutations. This presents challenges to treatment, as plasmapheresis may not be effective in those cases and complement inhibitors such as eculizumab should be used instead.

## OTHER TYPES OF APHERESIS

Other apheresis techniques may be employed in various other conditions. Although the available studies on these conditions are limited, precluding a systematic review, this section contains a narrative synthesis and expert opinion of the use of low-density lipoprotein apheresis in kidney disease, as we believe it is important to highlight their potential relevance.

### Lipoprotein apheresis

Low-density lipoprotein apheresis (LDL-A) is a blood purification therapy to remove low-density and very low-density lipoprotein, which was originally developed for primary hypercholesterolemia unresponsive to ordinary drug therapy [62]. See Supplemental Table S12 for an example protocol.

Lipoprotein apheresis (LDL-A) can be performed using the double filtration system, heparin-induced LDL precipitation, LDL adsorption through dextran sulfate, the DALI hemoperfusion system, and the Liposorber D system [63]. The heparin-based HELP system involves precipitating LDL by adding heparin to plasma and lowering the pH, with the precipitate removed via filtration. Another approach, DFPP, uses two filters to separate plasma from blood cells and selectively retain smaller components such as high-density lipoprotein (HDL) and albumin, while removing larger molecules such as LDL and Lp(a). The other approaches bind LDL based on negatively charged polyacrylate or dextran sulfate anions or antibodies to the lipoprotein [64]. Acute decreases in LDL-cholesterol after each procedure range from 60% to 80%, depending upon the volume of blood or plasma treated [65]. Guidelines from the Japanese Society of Nephrology include LDL-A as a treatment option for drug-resistant membranous nephropathy and FSGS, while guidelines by the Japanese Society for Pediatric Nephrology highlight the early use of PLEX and LDL-A as effective extra treatments for steroid-resistant syndrome [66, 67]. The persistence of nephrotic syndrome despite two lines of therapy increases the risk of kidney failure, with PLEX achieving remission (proteinuria between 0.3 and 3 g/d, associated with a serum albumin concentration >30 g/l) in 75% of post-transplant cases of nephrotic syndrome, suggesting the usage of LDL-A as a treatment option for refractory INS in native kidneys [68]. Studies showing the usage of LDL-A in FSGS are shown in Supplemental Table S13.

LDL-A has two mechanisms of action in treating nephrotic syndrome: the direct removal of lipoproteins that contain apolipoprotein-B (apoB) and the indirect enhancement of steroids and cyclosporine A (CsA) [69]. In clinical practice, the combination of LDL-A along with steroids and/or CsA is administered for optimal results [70]. Furthermore, it was discovered that higher success rates of remission were correlated with earlier initiation of LDL-A therapy [70]. In addition, Sannomiya *et al.* reported in a retrospective study that LDL-A administration to patients before surgery prevented the recurrence of FSGS following a kidney transplant in a study including five patients [71]. All five patients were given LDL-A before transplantation and demonstrated normal serum LDL levels after surgery, highlighting its efficacy. The prospective POLARIS study found that the short-term efficacy (proteinuria <1.0 g/day) rate at 1 month after LDL-A was 54.9% and the long-term remission (urine protein undetectable) rate at 2 years after treatment was 47.7% [72]. In addition, in patients with an eGFR ≥30 ml/min/1.73 m^2^, 26 (89.7%) of the 29 patients had recovered from NS, and 28 (96.6%) had avoided kidney failure at 2 years after treatment. However, in patients with severe decrease in kidney function (pre-treatment GFR <30 ml/min/1.73 m^2^), there was no significant improvement in eGFR [15.6 ± 7.5 ml/min/1.73 m^2^ for eGFR (*P* = .056)] [73].

LDL-A reduces the harmful lipid accumulation that leads to podocyte damage and glomerular sclerosis. By eliminating apoB-containing lipoproteins, podocyte function is protected by reducing the amount of oxidative stress and inflammation within the glomeruli. This intervention has been proposed to break the cycle of lipid-induced podocyte injury, proteinuria, and hypoalbuminemia. In refractory FSGS, LDL-A can reduce macrophage stimulation by ox-LDL, upregulate IL-10, decrease PCSK9 levels and IL-6, sCD40L, IL-31, increase vascular endothelial growth factor levels, and decrease thromboxane A2. Furthermore, LDL-A improves the absorption and efficacy of standard CsA and steroid therapy, boosting their anti-inflammatory effects and enhancing the treatment of FSGS patients [74]. Similarly, in minimal change disease, LDL-A may have benefit in reducing endothelin-1 levels, which could improve kidney hemodynamic [75].

Limiting LDL-A use in children >13.6 kg (30 lbs) and discontinuing ACE inhibitors before starting treatment is recommended, as 27.5% of patients report stress from the lengthy procedure and 34.8% experience significant pain during LDL-A, affecting their quality of life and highlighting the need for improved protocols to enhance compliance and management of paediatric patients [63, 76]. However, Shah *et al.* found that LDL-A treatment in a case series of children with recurrent FSGS demonstrated improvement in urinary protein to creatinine ratios with 57% (4/7) of the patients achieving complete remission (proteinuria <0.3 g/day) and 86% (6/7) of patients showed significant improvement in estimated GFR from time of LDL-A initiation to the time of discontinuation [77]. Using the Liposorber LA-15 system, the mean LDL pre-treatment was 188 mg/dl and post-treatment was 49 mg/dl (*P* < .001), cholesterol pre-treatment was 323 mg/dl and post-treatment was 124 mg/dl (*P* < .001), and triglycerides pre-treatment were 449 mg/dl and post-treatment were 212 mg/dl (*P* < .001), in five children, four of whom (80%) showed improvement in symptoms.

LDL-A may be used for minimal change disease refractory to other treatment and has shown to be beneficial in patients who have difficulty decreasing the steroid treatment dosage. Partial to complete remission has been found in 70%–95% of patients who underwent LDL-A.

Furthermore, LDL-A may be used in other lipid metabolism disorders, such as familial lecithin-cholesterol acyltransferase deficiency (FLD). FLD is characterized by impaired cholesterol esterification and reduced maturation of HDL particles [78]. Kidney failure remains a significant cause of mortality in FLD patients, likely due to lipoprotein X [79]. There is a need for more data in the paediatric population, as well as standardization of plasmapheresis dosage and duration.

### Limitations

Limitations of the study include an inability to compare between treatment modalities (plasmapheresis, PLEX, immunoadsorption) due to lack of data and differentiation in the included studies. Heterogeneity in therapeutic regimens including variations in adjunct immunosuppression, treatment duration, and plasmapheresis protocols probably influenced renal and haematologic outcomes across studies. Analysis was also limited due to small sample sizes, potentially limiting the reliability of these findings; however, they were included to highlight potential trends warranting future investigation in larger, adequately powered studies. In addition, some studies had a lack of kidney biopsy confirmation in patients, which may affect the results. Finally, there is variable follow-up between studies beyond the initial treatment course, which restricts our ability to assess sustained treatment efficacy, disease progression, and long-term patient outcomes.

## CONCLUSIONS

Plasmapheresis and related apheresis therapies have demonstrated promise in treating various kidney diseases, particularly in cases of autoantibody-mediated conditions such as anti-GBM disease, AAV, and FSGS. Although plasmapheresis shows promise, evidence in conditions such as lupus nephritis and IgA nephropathy is less robust, warranting further investigation. Moreover, while plasmapheresis is well-tolerated in many cases, adverse events such as hypocalcaemia and coagulopathies highlight the need for careful monitoring, particularly in paediatric populations. The choice of technique, either centrifugation or membrane filtration, plays a critical role in managing paediatric patients, given their distinct physiological characteristics. Overall, while plasmapheresis is a valuable tool in nephrology, ongoing research is necessary to refine its indications, improve patient outcomes, and reduce complications. Future studies, particularly multicentre trials and the development of global clinical registries, are essential to refine the therapeutic indications, optimize patient outcomes, and mitigate associated complications.

## STATEMENT OF ETHICS

This study protocol was reviewed, and ethics approval was not required as determined by the Northeast Ohio Medical University IRB.

## Supplementary Material

sfaf282_Supplemental_File

## Data Availability

The data that support the findings of this study are available from the corresponding author upon reasonable request.

## Notes

**Registration:** Prospero registration number: CRD42024598703
