# Supplementary material for: Indications and recent evidence for apheresis in children and adults with kidney diseases: a comprehensive review
Source: Clin Kidney J. 2025 Sep 10;18(10):sfaf282. doi: 10.1093/ckj/sfaf282 (PMC12511939; doi:10.1093/ckj/sfaf282)
Supplement: sfaf282_Supplemental_File [file sfaf282_supplemental_file.docx]

**Supplemental Material**

**Methods**

**Study Objectives**

The primary objective of this systematic review is to evaluate the efficacy, safety, and clinical outcomes associated with plasmapheresis and related apheresis therapies in treating various kidney diseases across paediatric and adult populations. We aim to determine remission rates, the incidence of kidney failure, and the prevalence of AEs following these treatments. Additionally, the study seeks to identify the specific clinical settings and patient subgroups where plasmapheresis may yield the most significant therapeutic benefits, thereby providing clearer, evidence-based guidance for its application in clinical practice.

**Search Strategy**

Following the Preferred Reporting Items for Systematic Reviews and Meta-Analysis (PRISMA) guidelines (**Supplemental Table 1**), we performed a comprehensive literature search in the following databases: “PubMed”, “Web of Science”, “Cochrane Library”, and “CINAHL”. The search terms incorporated the following: (("Acute Disease" AND "Glomerulonephritis") OR "acute glomerulonephritis" OR "acute glomerular nephritis" OR "Post-infectious glomerulonephritis" OR "postinfectious glomerulonephritis" OR "infection-related glomerulonephritis" OR "crescentic glomerulonephritis" OR "rapidly progressive glomerulonephritis" OR "Poststreptococcal glomerulonephritis” OR “focal segmental glomerulonephritis” OR “transplant glomerulonephritis”) AND ("Renal Replacement" OR "detoxification" OR "Renal Dialysis" OR "extracorporeal" OR "Plasmapheresis" OR "Plasma Exchange" OR "immunoadsorption" OR "Blood Component Removal" OR "Low-density lipoprotein apheresis" OR "Lipoprotein apheresis" OR "Lipoproteins" OR "Low-density lipoprotein apheresis") AND "English"[Language]. The search was restricted to studies published in English to ensure clarity in data interpretation and maintain consistency in the review process. The articles were screened for relevance and duplicate publications were removed. We also searched additional sources for relevant citations, including GreyNet International, SIGLE (The System for Information on Grey Literature in Europe), Open Grey, and Grey Literature Report. The AMSTAR checklist (**Supplemental Table 2**) and PICO table (**Supplemental Table 3**) are shown below.

**Study Selection and Review:**

The search encompassed all extracorporeal therapies used to treat acute GN such as, but not limited to, therapeutic plasma exchange (TPE), low-density lipoprotein (LDL) - apheresis, immunoadsorption, and dialysis, comparing these techniques to the first-line standard of treatment, administration of corticosteroids and immunosuppressive therapy without extracorporeal therapy. The study’s outcomes aim to evaluate the association between acute GN and patient’s length of hospital stay, mortality, development of end-stage kidney disease, associated comorbidities, and adverse events. Prospective, retrospective, case-controlled, and observational studies regarding plasmapheresis in adults and children were included. The included studies were classified as adult (>18 years of age) and paediatric (≤18 years of age) studies. Two independent reviewers examined the collected studies' titles, abstracts, and full texts (P.M. and E.C.). Any disagreements regarding inclusion criteria, study selection, or study quality rating were resolved via a consensus of the two reviewers or, if necessary, by an additional independent reviewer (R.R.). A complete list of studies included can be found in the second **Supplement B**.

**Data Extraction:**

The data extraction was recorded electronically using a standardized collection form, including title, authors, publication year, geography, study type, age, gender, dialysis requirement, remission, adverse events, and mortality. Additionally, we collected information on patient characteristics, such as demographics, clinical, and laboratory variables when available. Studies included randomized control trials, cohort studies, and case-control studies of patients of all ages and genders who had glomerulonephritis and underwent some form of extracorporeal therapy.

**Statistical Analysis**

The analysis included the different outcomes [remission, renal failure, dialysis requirement, mortality and adverse events] at the follow-up period. Heterogeneity across studies was quantified using the I^2^ statistic, and the I^2^ >50 % indicated significant heterogeneity. To determine the source of heterogeneity, sensitivity analyses were performed based on the indications. The fixed-effect analytical model was used to pool the results of studies with acceptable or no heterogeneity and random-effect model for studies with significant heterogeneity. Forest plot was used to visualize the mortality outcome in each study and the combined estimated outcome with their 95% CI. Publication bias was assessed graphically using funnel plots and Egger’s test. A p value ≤0.05 was considered for statistical significance. All statistical analyses were performed with MedCalc Statistical Software version 19.2.6 (MedCalc Software bv, Ostend, Belgium; https://www.medcalc.org; 2020).

**Included studies**

A total of 33 studies [12 conducted before 2015, 13 during 2015-2019 and 8 studies post 2019] were included. The total sample size of all the included studies was 1,363 ranging from 3 to 352 across the studies. The mean / median age of the included subjects ranged from 9 to 72 years and 59.1% of the subjects were males. The majority of the studies were retrospective cohort study [n=28 studies], followed by prospective cohort study [n=4] and randomized controlled trial [n=1]. The majority of the studies were conducted in Europe [n=13], followed by Asia [n=10], United States [n=8] and South America [n=1] and across multiple geographies [n=1]. The mean serum creatinine ranged from 0.8 to 9.6 mg/dl in the 5 studies reporting the data, mean estimated Glomerular filtration rate ranged from 9 to 91 mL/min/1.73m^2^ in the 10 studies reporting the data. A total of 12 studies included patients with a requirement for dialysis with a proportion of 47% [210/447]. The follow-up period ranged from 1 year to 4 years across the 23 studies reporting the data. With respect to the treatment, the patients received plasmapheresis in 31 studies [98.6% (1,312/1,330)], cyclophosphamide in 11 studies [64.9% (524/807)], methylprednisone in 8 studies [59.8% (354/592)], rituximab in 7 studies [20.4% (132/647)] and immunoadsorption in 3 studies [45.1% (23/51)]. The studies were analyzed collectively regardless of whether apheresis was used alone or in combination with other therapies. Based on the indications, 10 studies included subjects with AAV, 9 studies with renal transplant, 6 with anti-GBM, 6 with FSGS, 3 with RPGN and 2 with crescentic IgA nephropathy such that 3 studies had subjects with more than 1 indication.

**Sensitivity analysis**

The I^2^ value for sensitivity analyses for the outcomes were almost similar to that obtained by analyses based on the indications. Also, the pooled results obtained from the sensitivity analyses were within the 95% CI of the overall pooled outcomes, indicating that the results of this meta-analysis are robust enough.

The pooled proportion (95% CI) of remission was 60.65% (47.35% - 73.18%) [I^2^: 85.77% (78.66% - 90.51%); p<0.0001; 17 studies; N=420; **Supplemental Table 4; Supplemental Figure 1a**]. The Egger’s test [p=0.1318] and funnel plot [**Supplemental Figure 1b**] indicated the absence of publication bias. On sensitivity analysis based on the indications, the pooled proportion (95% CI) of remission ranged from 56.19% (35.71% - 75.62%) in renal transplant patients to 71.60% (62.68% - 79.42%) in ANCA-Associated Vasculitis patients [**Supplementary Table 9a**]. However, remission data were not included in the main document because they were aggregated from a heterogeneous group of diseases with varying definitions of remission, distinct indications for apheresis, different associated treatments, and variable prognoses.

The pooled proportion (95% CI) of renal failure was 26.36 % (17.38 % - 36.47%) [I^2^: 89.99% (85.71 % - 92.98%); p<0.0001; 18 studies, N=1,005; **Supplemental Table 5; Supplemental Figure 2a**] across all the included studies. The Egger’s test [p=0.7402] and funnel plot [**Supplemental Figure 2b**] indicated the absence of publication bias. On sensitivity analysis based on the indications, the pooled proportion (95% CI) of renal failure ranged from 18.87% (8.66% - 31.89%) in renal transplant patients to 62.93% (25.65% - 92.91%) in Anti-GBM patients [**Supplementary Table 9b**].

The pooled proportion (95% CI) of dialysis requirement was 30.43% (14.80% - 48.82%) [I^2^: 91.24% (87.06% - 94.07%); p<0.001; 14 studies; N=356; **Table 6; Figure 3a**]. The Egger’s test [p=0.6085] and funnel plot [**Supplemental Figure 3b**] indicated the absence of publication bias. On sensitivity analysis based on the indications, he pooled proportion (95% CI) of dialysis requirement ranged from 1.05% (0.00% - 10.17%) in renal transplant patients to 61.54 % (31.23 % - 87.59%) in RPGN and crescentic IgA Nephropathy patients [**Supplementary Table 9c**].

The pooled proportion (95% CI) of mortality was 10.86% (9.12 % - 12.81%) [I^2^: 48.42% (19.31 % - 67.03%); p=0.0028; 27 studies; N=1,114; **Supplemental Table 7; Supplemental Figure 4a**]. The Egger’s test [p=0.0696] and funnel plot [**Supplemental Figure 4b**] indicated the absence of publication bias. On sensitivity analysis based on the indications, the pooled proportion (95% CI) of mortality ranged from 2.10% (0.12% - 8.72%) in FSGS to 15.87% (6.93% - 27.59%) in anti-GBM patients [**Supplemental Table 9d**].

The pooled proportion (95% CI) of adverse events was 31.03% (12.78% - 53.05%) [I^2^: 92.93% (88.74% - 95.56%); p<0.0001; 9 studies; N=344; **Supplemental Table 8; Supplemental Figure 5a**]. The Egger’s test [p=0.1680] and funnel plot [**Supplemental Figure 5b**] indicated the absence of publication bias. On sensitivity analysis based on the indications, the pooled proportion (95% CI) adverse events ranged from 11.91% (0.78% - 42.92%) in FSGS to 68.83% (54.69% - 81.37%) in ANCA-Associated Vasculitis patients [**Supplemental Table 9e**]. Sensitivity analyses of dialysis requirement, mortality, adverse events, and remission are found in **Supplemental Table 9a-e**.

LDL-A was not included in the systematic review; instead, a summary point was generated based on a targeted review of available evidence and informal expert input. The methodology highlights the unique aspects of LDL-A as an apheresis modality and acknowledges the relative strength and limitations of the conclusions drawn.

The expert panel members were identified based on their expertise and involvement in the field, and the summary points were developed through discussions and consensus.

**Assessment of risk of bias in included studies:**

Two independent reviewers (P.M. and E.C.) graded each study for its level of evidence based on methodological quality, validity, and applicability using the Newcastle-Ottawa Scale (NOS) for quality assessment. A qualitative evaluation of the studies included is detailed in **Supplemental Table 10**. Observational studies with scores ≥7 points were considered of “good quality,” studies with 4-6 points were considered “fair quality.” Studies with ≤ 3 points were considered as “poor quality.” Additionally, certainty of evidence for each outcome was assessed using the Grading of Recommendations Assessment, Development and Evaluation (GRADE) framework. A qualitative evaluation of each pooled outcome is provided in Supplemental Table 14. This assessment rates the overall quality of evidence as high, moderate, low, or very low based on study design, risk of bias, inconsistency, and indirectness.

| **Supplemental Table 1:** PRISMA checklist | | | |
| --- | --- | --- | --- |
| **Section and Topic** | **Item #** | **Checklist item** | **Location where item is reported** |
| **TITLE** | | |  |
| Title | 1 | Identify the report as a systematic review. | Line 1, main manuscript |
| **ABSTRACT** | | |  |
| Abstract | 2 | See the PRISMA 2020 for Abstracts checklist. | Attached |
| **INTRODUCTION** | | |  |
| Rationale | 3 | Describe the rationale for the review in the context of existing knowledge. | Line 58 |
| Objectives | 4 | Provide an explicit statement of the objective(s) or question(s) the review addresses. | Line 60 |
| **METHODS** | | |  |
| Eligibility criteria | 5 | Specify the inclusion and exclusion criteria for the review and how studies were grouped for the syntheses. | Supplemental file |
| Information sources | 6 | Specify all databases, registers, websites, organisations, reference lists and other sources searched or consulted to identify studies. Specify the date when each source was last searched or consulted. | Supplemental file |
| Search strategy | 7 | Present the full search strategies for all databases, registers and websites, including any filters and limits used. | Supplemental file |
| Selection process | 8 | Specify the methods used to decide whether a study met the inclusion criteria of the review, including how many reviewers screened each record and each report retrieved, whether they worked independently, and if applicable, details of automation tools used in the process. | Supplemental file |
| Data collection process | 9 | Specify the methods used to collect data from reports, including how many reviewers collected data from each report, whether they worked independently, any processes for obtaining or confirming data from study investigators, and if applicable, details of automation tools used in the process. | Supplemental file |
| Data items | 10a | List and define all outcomes for which data were sought. Specify whether all results that were compatible with each outcome domain in each study were sought (e.g. for all measures, time points, analyses), and if not, the methods used to decide which results to collect. | Supplemental file |
|  | 10b | List and define all other variables for which data were sought (e.g. participant and intervention characteristics, funding sources). Describe any assumptions made about any missing or unclear information. | Supplemental file |
| Study risk of bias assessment | 11 | Specify the methods used to assess risk of bias in the included studies, including details of the tool(s) used, how many reviewers assessed each study and whether they worked independently, and if applicable, details of automation tools used in the process. | Supplemental file |
| Effect measures | 12 | Specify for each outcome the effect measure(s) (e.g. risk ratio, mean difference) used in the synthesis or presentation of results. | Supplemental file |
| Synthesis methods | 13a | Describe the processes used to decide which studies were eligible for each synthesis (e.g. tabulating the study intervention characteristics and comparing against the planned groups for each synthesis (item #5)). | Supplemental file |
|  | 13b | Describe any methods required to prepare the data for presentation or synthesis, such as handling of missing summary statistics, or data conversions. | Supplemental file |
|  | 13c | Describe any methods used to tabulate or visually display results of individual studies and syntheses. | Supplemental file |
|  | 13d | Describe any methods used to synthesize results and provide a rationale for the choice(s). If meta-analysis was performed, describe the model(s), method(s) to identify the presence and extent of statistical heterogeneity, and software package(s) used. | Supplemental file |
|  | 13e | Describe any methods used to explore possible causes of heterogeneity among study results (e.g. subgroup analysis, meta-regression). | Supplemental file |
|  | 13f | Describe any sensitivity analyses conducted to assess robustness of the synthesized results. | Supplemental file |
| Reporting bias assessment | 14 | Describe any methods used to assess risk of bias due to missing results in a synthesis (arising from reporting biases). | Supplemental file |
| Certainty assessment | 15 | Describe any methods used to assess certainty (or confidence) in the body of evidence for an outcome. | Supplemental file |
| **RESULTS** | | |  |
| Study selection | 16a | Describe the results of the search and selection process, from the number of records identified in the search to the number of studies included in the review, ideally using a flow diagram. | Supplemental file |
|  | 16b | Cite studies that might appear to meet the inclusion criteria, but which were excluded, and explain why they were excluded. | Supplemental file |
| Study characteristics | 17 | Cite each included study and present its characteristics. |  |
| Risk of bias in studies | 18 | Present assessments of risk of bias for each included study. |  |
| Results of individual studies | 19 | For all outcomes, present, for each study: (a) summary statistics for each group (where appropriate) and (b) an effect estimate and its precision (e.g. confidence/credible interval), ideally using structured tables or plots. | Supplemental file |
| Results of syntheses | 20a | For each synthesis, briefly summarise the characteristics and risk of bias among contributing studies. | Supplemental file |
|  | 20b | Present results of all statistical syntheses conducted. If meta-analysis was done, present for each the summary estimate and its precision (e.g. confidence/credible interval) and measures of statistical heterogeneity. If comparing groups, describe the direction of the effect. | Supplemental file |
|  | 20c | Present results of all investigations of possible causes of heterogeneity among study results. | Supplemental file |
|  | 20d | Present results of all sensitivity analyses conducted to assess the robustness of the synthesized results. | Supplemental file |
| Reporting biases | 21 | Present assessments of risk of bias due to missing results (arising from reporting biases) for each synthesis assessed. | Supplemental file |
| Certainty of evidence | 22 | Present assessments of certainty (or confidence) in the body of evidence for each outcome assessed. | Supplemental file |
| **DISCUSSION** | | |  |
| Discussion | 23a | Provide a general interpretation of the results in the context of other evidence. | Line 73 |
|  | 23b | Discuss any limitations of the evidence included in the review. | Line 75 |
|  | 23c | Discuss any limitations of the review processes used. | Supplemental file |
|  | 23d | Discuss implications of the results for practice, policy, and future research. | Line 350 |
| **OTHER INFORMATION** | | |  |
| Registration and protocol | 24a | Provide registration information for the review, including register name and registration number, or state that the review was not registered. | Stated |
|  | 24b | Indicate where the review protocol can be accessed, or state that a protocol was not prepared. | Supplemental file |
|  | 24c | Describe and explain any amendments to information provided at registration or in the protocol. | N/A |
| Support | 25 | Describe sources of financial or non-financial support for the review, and the role of the funders or sponsors in the review. | See title page |
| Competing interests | 26 | Declare any competing interests of review authors. | See title page |
| Availability of data, code and other materials | 27 | Report which of the following are publicly available and where they can be found: template data collection forms; data extracted from included studies; data used for all analyses; analytic code; any other materials used in the review. | Supplemental file |

*From:*  Page MJ, McKenzie JE, Bossuyt PM, Boutron I, Hoffmann TC, Mulrow CD, et al. The PRISMA 2020 statement: an updated guideline for reporting systematic reviews. BMJ 2021;372:n71. doi: 10.1136/bmj.n71

| **Section and Topic** | **Item #** | **Checklist item** | **Reported (Yes/No)** |
| --- | --- | --- | --- |
| **TITLE** | | |  |
| Title | 1 | Identify the report as a systematic review. | Yes |
| **BACKGROUND** | | |  |
| Objectives | 2 | Provide an explicit statement of the main objective(s) or question(s) the review addresses. | Yes |
| **METHODS** | | |  |
| Eligibility criteria | 3 | Specify the inclusion and exclusion criteria for the review. | Yes |
| Information sources | 4 | Specify the information sources (e.g. databases, registers) used to identify studies and the date when each was last searched. | Yes |
| Risk of bias | 5 | Specify the methods used to assess risk of bias in the included studies. | Yes |
| Synthesis of results | 6 | Specify the methods used to present and synthesise results. | Yes |
| **RESULTS** | | |  |
| Included studies | 7 | Give the total number of included studies and participants and summarise relevant characteristics of studies. | Yes |
| Synthesis of results | 8 | Present results for main outcomes, preferably indicating the number of included studies and participants for each. If meta-analysis was done, report the summary estimate and confidence/credible interval. If comparing groups, indicate the direction of the effect (i.e. which group is favoured). | Yes |
| **DISCUSSION** | | |  |
| Limitations of evidence | 9 | Provide a brief summary of the limitations of the evidence included in the review (e.g. study risk of bias, inconsistency and imprecision). | Yes |
| Interpretation | 10 | Provide a general interpretation of the results and important implications. | Yes |
| **OTHER** | | |  |
| Funding | 11 | Specify the primary source of funding for the review. | Yes |
| Registration | 12 | Provide the register name and registration number. | Yes |

*From:*  Page MJ, McKenzie JE, Bossuyt PM, Boutron I, Hoffmann TC, Mulrow CD, et al. The PRISMA 2020 statement: an updated guideline for reporting systematic reviews. BMJ 2021;372:n71. doi: 10.1136/bmj.n71

**Supplemental Table 2:** AMSTAR Checklist

| 1. Did the research questions and inclusion criteria for the review include the components of PICO? | | |
| --- | --- | --- |
| Population**-Yes** | Timeframe for Follow-up-**No** |  |
| Intervention**-Yes** |  |  |
| Comparator group**-Yes** |  |  |
| Outcome**-Yes** |  |  |
| 2. Did the report of the review contain an explicit statement that the review methods were established prior to the conduct of the review and did the report justify any significant deviations from the protocol? | | |
| Review Question | a meta-analysis/synthesis plan, if appropriate, and -**Yes** |  |
| a search strategy **-Yes** | a plan for investigating causes of heterogeneity -**Yes** |  |
| inclusion/exclusion criteria **-Yes** | justification for any deviations from the protocol **-Yes** |  |
| a risk of bias assessment **-Yes** |  |  |
| 3. Did the review authors explain their selection of the study designs for inclusion in the review? **Yes** | | |
| OR Explanation for including both RCTs and NRSI |  |  |
| 4. Did the review authors use a comprehensive literature search strategy? | | |
| searched at least two databases (relevant to the research question) --**Yes** | Searched the reference lists/bibliographies of included studies   Searched trial/study registries included/consulted content experts in the field-**Yes** |  |
| provided keyword and/or   search strategy **-Yes** | where relevant, searched for grey literature **-Yes** |  |
| justified publication restrictions **-Yes** | conducted search within 24 months of completion of the review -**Yes** |  |
|  | Searched the reference lists/bibliographies of included studies   Searched trial/study registries included/consulted content experts in the field **-Yes** |  |
| 5. Did the review authors perform study selection in duplicate? | | |
| At least two reviewers independently agreed on selection of eligible studies and achieved consensus on which studies to include**-Yes** |  |  |
| 6. Did the review authors perform data extraction in duplicate? | | |
| at least two reviewers achieved consensus on which data to extract from included studies **-Yes** |  |  |
| 7. Did the review authors provide a list of excluded studies and justify the exclusions? | | |
| Provided a list of all potentially relevant studies that were read in full-text form but excluded from the review**-No** | Justified the exclusion from the review of each potentially relevant study-**No** |  |
| 8. Did the review authors describe the included studies in adequate detail? | | |
| described populations - | described population in detail **-Yes** |  |
| described interventions **-Yes** | described intervention in detail (including doses where relevant) **-Yes** |  |
| described comparators **-Yes** |  |  |
| described outcomes **-Yes** | described study’s setting **-Yes** |  |
| described research designs **-Yes** | timeframe for follow-up **-Yes** |  |
| 9. Did the review authors use a satisfactory technique for assessing the risk of bias (RoB) in individual studies that were included in the review? | | |
| **RCT** |  |  |
| unconcealed allocation, and lack of blinding of patients and **-Yes** | allocation sequence that was not truly random, and **-Yes** |  |
| assessors when assessing outcomes (unnecessary for objective outcomes such as all-cause mortality) **-Yes** | selection of the reported result from among multiple measurements or analyses of a specified outcome **-Yes** |  |
| NRSI |  |  |
| from confounding bias**-Yes** | methods used to ascertain exposures and outcomes, and **-Yes** |  |
| from selection bias and from selection bias | selection of the reported result from among multiple measurements or analyses of a specified outcome **-Yes** |  |
| 10. Did the review authors report on the sources of funding for the studies included in the review? | | |
| Must have reported on the sources of funding for individual studies included, but study authors did not report it also qualifies **-Yes** |  |  |
| If meta-analysis was performed, did the review authors use appropriate methods to combine the statistical results? | | |
| **RCT** |  |  |
| The authors justified combining the data in a meta-analysis **-Yes** |  |  |
| They used an appropriate weighted technique to combine study results and adjust for heterogeneity if present. **-Yes** |  |  |
| AND investigated the causes of any heterogeneity**-Yes** |  |  |
| **NRSI** |  |  |
| The authors justified combining the data in a meta-analysis **-Yes** |  |  |
| AND they used an appropriate weighted technique to combine study results, adjusting for heterogeneity if present**-Yes** |  |  |
| AND they statistically combined effect estimates from NRSI that were adjusted for confounding, rather than combining raw data, or justified combining raw data when adjusted effect estimates were not available **-Yes** |  |  |
| AND they reported separate summary estimates for RCTs and NRSI separately when both were included in the review **-Yes** |  |  |
| 11. If meta-analysis was performed, did the review authors assess the potential impact of RoB in individual studies on the results of the meta-analysis or other evidence synthesis? | | |
| OR, if the pooled estimate was based on RCTs and/or NRSI at variable **-Yes** |  |  |
| 12. Did the review authors account for RoB in individual studies when interpreting/ discussing the results of the review? | | |
| If RCTs with moderate or high RoB or NRSI were included, the review provided a discussion of the likely impact of RoB on the results **-Yes** |  |  |
| 13. Did the review authors provide a satisfactory explanation for, and discussion of, any heterogeneity observed in the results of the review? | | |
| OR if heterogeneity was present, the authors performed an investigation of sources of any heterogeneity in the results and discussed the impact of this on the results of the review **-Yes** |  |  |
| 14. If they performed quantitative synthesis, did the review authors carry out an adequate investigation of publication bias (small study bias) and discuss its likely impact on the results of the review? | | |
| Performed graphical or statistical tests for publication bias and discussed the likelihood and magnitude of impact of publication bias **-Yes** |  |  |
| 15. Did the review authors report any potential sources of conflict of interest, including any funding they received for conducting the review? | | |
| The authors reported no competing interests**-Yes** |  |  |

**Supplemental Table 3:** PICO table

| Population (P) | Paediatric and adult patients with kidney diseases (e.g., ANCA-associated vasculitis, FSGS, RPGN, lupus nephritis, anti-GBM disease, kidney transplant cases). |
| --- | --- |
| Intervention (I) | Plasmapheresis or related apheresis therapies (e.g., therapeutic plasma exchange, LDL-apheresis). |
| Comparison (C) | Standard treatments without plasmapheresis or alternative interventions (e.g., corticosteroids, immunosuppressives alone). |
| Outcomes (O) | Effectiveness (remission rates, reduction in kidney failure or dialysis requirement), safety (adverse events), mortality, hospital stay duration, and comorbidities. |
|  |  |

**Supplemental Table 4:** Meta-analysis of remission

| Study | Remission  [n] | Sample  size [N] | Proportion  (95% CI) | Random Weight (%) |
| --- | --- | --- | --- | --- |
| Zhang et al., 2023 | 1 | 3 | 33.33 (0.84 - 90.57) | 3.76 |
| Restrepo et al., 2022 | 15 | 17 | 88.23 (63.55 - 98.54) | 6.2 |
| Gulati et al., 2021 | 48 | 64 | 75.00 (62.60 - 84.98) | 7.16 |
| Moura et al., 2020 | 36 | 51 | 70.58 (56.17 - 82.51) | 7.05 |
| Campise et al., 2019 | 13 | 21 | 61.90 (38.43 - 81.89) | 6.41 |
| Ersan et al., 2019 | 2 | 4 | 50.00 (6.759 - 93.24) | 4.19 |
| Koutroutsos et al., 2019 | 9 | 10 | 90.00 (55.49 - 99.74) | 5.55 |
| Raina et al., 2019 | 4 | 5 | 80.00 (28.35 - 99.49) | 4.53 |
| Alasfar et al., 2018 | 23 | 66 | 34.84 (23.52 - 47.57) | 7.17 |
| Verghese et al., 2018 | 9 | 57 | 15.78 (7.483 - 27.86) | 7.11 |
| Muso et al., 2015 | 12 | 28 | 42.85 (24.46 - 62.82) | 6.67 |
| Paglialonga et al., 2015 | 9 | 13 | 69.23 (38.57 - 90.90) | 5.89 |
| Park et al., 2014 | 7 | 27 | 25.92 (11.11 - 46.28) | 6.64 |
| Samanci et al., 2014 | 5 | 6 | 83.33 (35.87 - 99.57) | 4.8 |
| Straatmann et al., 2014 | 7 | 7 | 100.0 (59.03 - 00.00) | 5.04 |
| Gonzalez et al., 2011 | 15 | 34 | 44.11 (27.18 - 62.11) | 6.81 |
| Fuentes et al., 2010 | 6 | 7 | 85.71 (42.12 - 99.63) | 5.04 |
| Total (random effects) | 221 | 420 | 60.65 (47.35 - 73.18) | 100 |

**Supplemental Figure 1a:** Forest plot of the meta-analysis of remission. The lower diamond in the graph represents the pooled estimate

**Supplemental Figure 1b:** Funnel plot for showing publication bias for remission.

**Supplemental Table 5:** Meta-analysis of renal failure

| Study | Renal failure  [n] | Sample  size [N] | Proportion  (95% CI) | Random  Weight (%) |
| --- | --- | --- | --- | --- |
| Jayne et al., 2022 | 67 | 352 | 19.03 (15.06 - 23.53) | 6.82 |
| Wang et al., 2022 | 21 | 37 | 56.75 (39.48 - 72.90) | 6.04 |
| Gulati et al., 2021 | 12 | 64 | 18.75 (10.08 - 30.46) | 6.38 |
| Moura et al., 2020 | 17 | 51 | 33.33 (20.75 - 47.92) | 6.26 |
| Campise et al., 2019 | 4 | 21 | 19.04 (5.44 - 41.90) | 5.53 |
| Koutroutsos et al., 2019 | 1 | 10 | 10.00 (0.25 - 44.50) | 4.6 |
| Nishimura et al., 2019 | 2 | 12 | 16.66 (2.08 - 48.41) | 4.85 |
| Alasfar et al., 2018 | 8 | 66 | 12.12 (5.38 - 22.49) | 6.4 |
| Kouri and Andreoli et al., 2017 | 0 | 5 | 0.00 (0.00 - 52.18) | 3.59 |
| Xie et al., 2016 | 7 | 12 | 58.33 (27.66 - 84.83) | 4.85 |
| Luna et al., 2015 | 29 | 152 | 19.07 (13.16 - 26.23) | 6.69 |
| Biesenbach et al., 2014 | 4 | 10 | 40.00 (12.15 - 73.76) | 4.6 |
| Park et al., 2014 | 2 | 27 | 7.40 (0.91 - 24.29) | 5.78 |
| Sinha et al., 2012 | 2 | 8 | 25.00 (3.18 - 65.08) | 4.28 |
| Siomou et al., 2012 | 0 | 10 | 0.00 (0.00 - 30.85) | 4.6 |
| Cui et al., 2011 | 60 | 76 | 78.94 (68.07 - 87.46) | 6.46 |
| Gonzalez et al., 2011 | 5 | 34 | 14.70 (4.95 - 31.05) | 5.98 |
| Gungor et al., 2011 | 26 | 58 | 44.82 (31.74 - 58.45) | 6.33 |
| Total (random effects) | 267 | 1,005 | 26.36 (17.38 - 36.47) | 100 |

**Supplemental Figure 2a:** Forest plot of the meta-analysis of renal failure. The lower diamond in the graph represents the pooled estimate **Supplemental Figure 2b:** Funnel plot for showing publication bias in renal failure

**Supplemental Table 6:** Meta-analysis of dialysis requirement

| Study | Dialysis requirement [n] | Sample  size [N] | Proportion (95% CI) | Random Weight (%) |
| --- | --- | --- | --- | --- |
| Zhang et al., 2023 | 2 | 3 | 66.66 (9.43 - 99.16) | 5.46 |
| Morel et al., 2022 | 20 | 76 | 26.31 (16.87 - 37.67) | 8.36 |
| Wang et al., 2022 | 34 | 37 | 91.89 (78.09 - 98.29) | 8.11 |
| Henriksson et al., 2021 | 9 | 96 | 9.37 (4.37 - 17.05) | 8.41 |
| Moura et al., 2020 | 20 | 51 | 39.21 (25.84 - 53.88) | 8.24 |
| Kouri and Andreoli et al., 2017 | 0 | 5 | 0.00 (0.00 - 52.18) | 6.22 |
| Xie et al., 2016 | 6 | 12 | 50.00 (21.09 - 78.90) | 7.31 |
| Touzot et al., 2015 | 1 | 5 | 20.00 (0.50 - 71.64) | 6.22 |
| Biesenbach et al., 2014 | 3 | 10 | 30.00 (6.67 - 65.24) | 7.12 |
| Samanci et al., 2014 | 2 | 6 | 33.33 (4.32 - 77.72) | 6.47 |
| Straatmann et al., 2014 | 0 | 7 | 0.00 (0.00 - 40.96) | 6.68 |
| Sinha et al., 2012 | 2 | 8 | 25.00 (3.185 - 65.08) | 6.85 |
| Srivastava et al., 2012 | 4 | 6 | 66.66 (22.27 - 95.67) | 6.47 |
| Gonzalez et al., 2011 | 0 | 34 | 0.00 (0.00 - 10.28) | 8.08 |
| Total (random effects) | 103 | 356 | 30.43 (14.80 - 48.82) | 100 |

**Supplemental Figure 3a:** Forest plot of the meta-analysis of dialysis requirement. The lower diamond in the graph represents the pooled estimate

**Supplemental Figure 3b:** Funnel plot for showing publication bias for dialysis requirement

**Supplemental Table 7:** Meta-analysis of mortality

| Study | Mortality  [n] | Sample  size [N] | Proportion  (95% CI) | Fixed Weight (%) |
| --- | --- | --- | --- | --- |
| Zhang et al., 2023 | 0 | 3 | 0 (0.00 - 70.76) | 0.35 |
| Jayne et al., 2022 | 46 | 352 | 13.06 (9.72 - 17.04) | 30.94 |
| Morel et al., 2022 | 4 | 76 | 5.26 (1.45 - 12.93) | 6.75 |
| Restrepo et al., 2022 | 1 | 17 | 5.88 (0.14 - 28.68) | 1.58 |
| Wang et al., 2022 | 2 | 37 | 5.40 (0.66 - 18.19) | 3.33 |
| Gulati et al., 2021 | 4 | 64 | 6.25 (1.72 - 15.23) | 5.7 |
| Henriksson et al., 2021 | 7 | 96 | 7.29 (2.98 - 14.44) | 8.5 |
| Moura et al., 2020 | 4 | 51 | 7.84 (2.17 - 18.88) | 4.56 |
| Campise et al., 2019 | 1 | 21 | 4.76 (0.12 - 23.81) | 1.93 |
| Ersan et al., 2019 | 0 | 4 | 0.00 (0.00 - 60.23) | 0.44 |
| Koutroutsos et al., 2019 | 1 | 10 | 10.00 (0.25 - 44.50) | 0.96 |
| Nishimura et al., 2019 | 3 | 12 | 25.00 (5.48 - 57.18) | 1.14 |
| Raina et al., 2019 | 0 | 5 | 0.00 (0.00 - 52.18) | 0.53 |
| Kouri and Andreoli et al., 2017 | 0 | 5 | 0.00 (0.00 - 52.18) | 0.53 |
| Xie et al., 2016 | 1 | 12 | 8.33 (0.21 - 38.48) | 1.14 |
| Luna et al., 2015 | 18 | 152 | 11.84 (7.17 - 18.06) | 13.41 |
| Muso et al., 2015 | 0 | 28 | 0.00 (0.00 - 12.34) | 2.54 |
| Paglialonga et al., 2015 | 0 | 13 | 0.00 (0.00 - 24.70) | 1.23 |
| Touzot et al., 2015 | 0 | 5 | 0.00 (0.00 - 52.18) | 0.53 |
| Biesenbach et al., 2014 | 1 | 10 | 10.00 (0.25 - 44.50) | 0.96 |
| Samanci et al., 2014 | 0 | 6 | 0.00 (0.00 - 45.92) | 0.61 |
| Zhang et al., 2014 | 6 | 28 | 21.42 (8.29 - 40.95) | 2.54 |
| Sinha et al., 2012 | 0 | 8 | 0 (0.000 - 36.94) | 0.79 |
| Siomou et al., 2012 | 0 | 10 | 0 (0.000 - 30.85) | 0.96 |
| Srivastava et al., 2012 | 0 | 6 | 0 (0.000 - 45.92) | 0.61 |
| Cui et al., 2011 | 24 | 76 | 31.57 (21.38 - 43.25) | 6.75 |
| Fuentes et al., 2010 | 0 | 7 | 0 (0.00 - 40.96) | 0.7 |
| Total (fixed effects) | 123 | 1,114 | 10.86 (9.12 - 12.81) | 100 |

**Supplemental Figure 4a:** Forest plot of the meta-analysis of mortality. The lower diamond in the graph represents the pooled estimate

**Supplemental Figure 4b:** Funnel plot for showing publication bias for mortality

**Supplemental Table 8:** Meta-analysis of adverse events

| Study | Adverse Events [n] | Sample  size [N] | Proportion (95% CI) | Random Weight (%) |
| --- | --- | --- | --- | --- |
| Restrepo et al., 2022 | 6 | 17 | 35.29 (14.21 - 61.67) | 11.51 |
| Gulati et al., 2021 | 49 | 64 | 76.56 (64.30 - 86.24) | 12.61 |
| Ersan et al., 2019 | 1 | 4 | 25.00 (0.63 - 80.58) | 8.75 |
| Luna et al., 2015 | 95 | 152 | 62.50 (54.29 - 70.21) | 12.89 |
| Touzot et al., 2015 | 1 | 5 | 20.00 (0.505 - 71.64) | 9.26 |
| Biesenbach et al., 2014 | 1 | 10 | 10.00 (0.253 - 44.50) | 10.68 |
| Samanci et al., 2014 | 0 | 6 | 0.00 (0.00 - 45.92) | 9.67 |
| Zhang et al., 2014 | 9 | 28 | 32.14 (15.87 - 52.35) | 12.06 |
| Gungor et al., 2011 | 4 | 58 | 6.89 (1.91 - 16.72) | 12.57 |
| Total (random effects) | 166 | 344 | 31.03 (12.78 - 53.05) | 100 |

**Supplemental Figure 5a:** Forest plot of the meta-analysis of adverse events. The lower diamond in the graph represents the pooled estimate

**Supplemental Figure 5b:** Funnel plot for showing publication bias for adverse events

**Supplementary table 9a:** Sensitivity analysis of Remission based on the indications

| **Indication** | **Number of studies** | **Sample size** | **Proportion**  **(95% CI)** | **I^2^ (95% CI), p value** | **Egger’s test**  **(p value)** |
| --- | --- | --- | --- | --- | --- |
| ANCA-Associated Vasculitis | 3 | 118 | 71.60%  (62.68% - 79.42%) | 15.22% (0.00% - 97.16%); p=0.3074 | 0.1566 |
| Renal transplant | 8 | 239 | 56.19%  (35.71% - 75.62%) | 89.95% (82.59% - 94.20%); p<0.0001 | 0.0061 |
| Anti-GBM | Not reported | | | | |
| FSGS | Not reported | | | | |
| RPGN and Crescentic IgA Nephropathy | 2 | 34 | 66.97%  (49.34 % - 81.69%) | 12.86% (0.00% - 25.06%); p=0.2841 | 0.9142 |

**Supplementary table 9b:** Sensitivity analysis of renal failure based on the indications

| **Indication** | **Number of studies** | **Sample size** | **Proportion**  **(95% CI)** | **I^2^ (95% CI), p value** | **Egger’s test**  **(p value)** |
| --- | --- | --- | --- | --- | --- |
| ANCA-Associated Vasculitis | 7 | 646 | 19.64 %  (16.66% - 22.90%) | 45.43% (0.0 % - 77.02%); p=0.0885 | 0.5269 |
| Renal transplant | 6 | 216 | 18.87%  (8.66% - 31.89%) | 78.84% (53.59% - 90.36%); p=0.0003 | 0.5544 |
| Anti-GBM | 2 | 86 | 62.93%  (25.65% - 92.91%) | 83.44% (31.20% - 96.01%); p=0.0140 | <0.0001 |
| FSGS | Not reported | | | | |
| RPGN and Crescentic IgA Nephropathy | 3 | 57 | 52.38%  (39.07% - 65.43%) | 28.06% (0.00% - 97.59%); p=0.2491 | 0.4986 |

**Supplementary table 9c:** Sensitivity analysis of dialysis requirement based on the indications

| **Indication** | **Number of studies** | **Sample size** | **Proportion**  **(95% CI)** | **I^2^ (95% CI), p value** | **Egger’s test**  **(p value)** |
| --- | --- | --- | --- | --- | --- |
| ANCA-Associated Vasculitis | 5 | 231 | 23.97%  (10.12% - 41.46%) | 83.58% (62.82% - 92.75%); p=0.0001 | 0.6835 |
| Renal transplant | 2 | 41 | 1.05%  (0.00% - 10.17%) | 0.00% (0.00% - 0.00%); p=0.6250 | <0.0001 |
| Anti-GBM | 3 | 21 | 39.11%  (20.06% - 60.95%) | 25.89% (0.00% - 97.51%); p=0.2594 | 0.8755 |
| FSGS |  |  |  |  |  |
| RPGN and Crescentic IgA Nephropathy | 4 | 74 | 61.54 %  (31.23 % - 87.59%) | 85.24% (63.48% - 94.03%); p=0.0001 | 0.0014 |

**Supplementary table 9d:** Sensitivity analysis of mortality based on the indications

| **Indication** | **Number of studies** | **Sample size** | **Proportion**  **(95% CI)** | **I^2^ (95% CI), p value** | **Egger’s test**  **(p value)** |
| --- | --- | --- | --- | --- | --- |
| ANCA-Associated Vasculitis | 10 | 821 | 10.59%  (8.58% - 12.88%) | 21.51% (0.00% - 61.53%); p=0.2450 | 0.2209 |
| Renal transplant | 3 | 48 | 8.45%  (2.50% - 19.67%) | 0.00% (0.00% - 82.22%); p=0.828 | 0.0030 |
| Anti-GBM | 6 | 152 | 15.87%  (6.93% - 27.59%) | 59.97% (1.72 % - 83.69%); p=0.0287 | 0.0336 |
| FSGS | 6 | 63 | 2.10%  (0.12% - 8.72%) | 0.00% (0.00 % - 0.00%); p=0.9844 | <0.0001 |
| RPGN and Crescentic IgA Nephropathy | 5 | 91 | 6.51%  (2.48% - 13.44%) | 0% (0.00% - 74.85%); p=0.5391 | 0.9142 |

**Supplementary table 9e:** Sensitivity analysis of adverse event based on the indications

| **Indication** | **Number of studies** | **Sample size** | **Proportion**  **(95% CI)** | **I^2^ (95% CI), p value** | **Egger’s test**  **(p value)** |
| --- | --- | --- | --- | --- | --- |
| ANCA-Associated Vasculitis | 2 | 216 | 68.83%  (54.69% - 81.37%) | 75.57% (0.00% - 94.46%); p=0.0430 | <0.0001 |
| Renal transplant | 2 | 75 | 18.82%  (0.84% - 52.00%) | 86.4% (45.95% - 96.58%); p=0.0067 | <0.0001 |
| Anti-GBM | 4 | 70 | 31.83%  (21.48% - 43.69%) | 13.13% (0.00 % - 88.79%); p=0.3268 | 0.2907 |
| FSGS | 2 | 10 | 11.91%  (0.78% - 42.92%) | 40.76% (0.00 % - 60.05%); p=0.1939 | <0.0001 |
| RPGN and Crescentic IgA Nephropathy | 2 | 34 | 40.62%  (5.19 % - 83.49%) | 87.46% (51.18% - 96.78%); p=0.0047 | 0.1925 |

**Supplemental Figure 6:** PRISMA diagram

**Identification of studies via other methods**

**Identification of studies via databases and registers**

Records identified from:

Citation searching (n = 5)

Records removed *before screening*:

Duplicate records removed (n = 408)

Records marked as ineligible by automation tools (n = 294)

Records identified from:

Databases (PubMed - 647 results

Web of Science - 547 results

Cochrane Library - 19 reviews, 172 trials

CINAHL - 283 results)

**Identification**

Records screened

(n = 555 )

Records excluded

(n = 390)

Reports not retrieved

(n = 0)

Reports sought for retrieval

(n = 5)

Reports sought for retrieval

(n = 165)

Reports not retrieved

(n = 0)

**Screening**

Reports excluded:

Did not meet inclusion criteria (n = 35)

Invalid Study Design (n = 32)

Before 2010 (n = 16)

Background Article (n = 16)

Invalid Outcome Measures (n = 14)

Invalid therapy (n = 13)

Reports assessed for eligibility

(n = 5)

Reports excluded:

(n = 0)

Reports assessed for eligibility

(n = 165)

Studies included in review

(n = 33)

**Included**

**Supplemental Table 10:** Newcastle-Ottawa Risk of Bias analysis

|  | **Author** | Representativeness of exposed cohort | Selection of the non-exposed cohort | Ascertainment of exposure | Demonstration that outcome of interest was not present at start of study | Comparability of cohorts based on the design or analysis | Assessment of outcome | Was follow-up long enough for out-comes to occur | Adequacy of follow up cohorts | Quality score |
| --- | --- | --- | --- | --- | --- | --- | --- | --- | --- | --- |
| Double filtration plasmapheresis for children with different types of critical kidney diseases: a single-centre retrospective cohort study | Zhang et al., 2023 | 1 | 0 | 1 | 1 | 0 | 1 | 1 | 0 | 5 |
| Plasma exchange and glucocorticoids to delay death or end-stage renal disease in anti-neutrophil cytoplasm antibody-associated vasculitis: PEXIVAS non-inferiority factorial RCT | Jayne et al., 2022 | 1 | 1 | 1 | 1 | 1 | 1 | 1 | 1 | 8 |
| Management of severe renal disease in anti-neutrophil-cytoplasmic-antibody-associated vasculitis: the place of rituximab and plasma exchange? | Morel et al., 2022 | 1 | 1 | 1 | 1 | 1 | 1 | 1 | 1 | 8 |
| Treatment of post-transplant recurrent FSGS in children using plasmapheresis and augmentation of immunosuppression | Restrepo et al., 2022 | 1 | 0 | 1 | 1 | 0 | 1 | 1 | 0 | 5 |
| Efficacy of plasma exchange in severe crescentic IgA nephropathy: a multicentred, cohort study | Wang et al., 2022 | 1 | 1 | 1 | 1 | 1 | 1 | 1 | 1 | 8 |
| Combination treatment with rituximab, low-dose cyclophosphamide and plasma exchange for severe antineutrophil cytoplasmic antibody-associated vasculitis | Gulati et al., 2021 | 1 | 0 | 1 | 1 | 0 | 1 | 1 | 1 | 6 |
| Analyses of registry data of patients with anti-GBM and antineutrophil cytoplasmatic antibody-associated (ANCA) vasculitis treated with or without therapeutic apheresis | Henriksson et al., 2021 | 1 | 1 | 1 | 1 | 1 | 1 | 1 | 1 | 8 |
| Efficacy of Rituximab and Plasma Exchange in Antineutrophil Cytoplasmic Antibody-Associated Vasculitis with Severe Kidney Disease | Moura et al., 2020 | 1 | 1 | 1 | 1 | 0 | 1 | 1 | 1 | 7 |
| Clinical Outcomes of Prophylactic and Therapeutic Plasmapheresis in Adult Deceased-Donor Kidney Transplant Recipients with Primary Focal Segmental Glomerulosclerosis | Campise et al., 2019 | 0 | 0 | 1 | 1 | 1 | 0 | 1 | 1 | 5 |
| Therapeutic Plasma Exchange in Renal Diseases: A Three-Year Retrospective Analysis | Ersan et al., 2019 | 1 | 0 | 1 | 1 | 0 | 1 | 1 | 1 | 6 |
| Successful management of post-transplant focal segmental glomerulosclerosis with therapeutic plasma exchange and rituximab | Koutroutsos et al., 2019 | 1 | 0 | 1 | 1 | 0 | 1 | 1 | 1 | 6 |
| Efficacy of Plasma Exchange in Anti-Neutrophil Cytoplasmic Antibody-Associated Vasculitis | Nishimura et al., 2019 | 1 | 1 | 1 | 1 | 0 | 1 | 1 | 1 | 7 |
| Dextran-Sulfate Plasma Adsorption Lipoprotein Apheresis in Drug Resistant Primary Focal Segmental Glomerulosclerosis Patients: Results from a Prospective, Multicentre, Single-Arm Intervention Study | Raina et al., 2019 | 1 | 1 | 1 | 1 | 0 | 1 | 1 | 1 | 7 |
| Rituximab and Therapeutic Plasma Exchange in Recurrent Focal Segmental Glomerulosclerosis Postkidney Transplantation | Alasfar et al., 2018 | 1 | 1 | 1 | 1 | 1 | 1 | 1 | 1 | 8 |
| The effect of peri-transplant plasmapheresis in the prevention of recurrent FSGS | Verghese et al., 2018 | 1 | 1 | 1 | 1 | 1 | 1 | 1 | 1 | 8 |
| Clinical presentation and outcome of paediatric ANCA-associated glomerulonephritis | Kouri and Andreoli et al., 2017 | 1 | 1 | 1 | 1 | 0 | 1 | 1 | 1 | 7 |
| Plasma Exchange as an Adjunctive Therapy for Crescentic IgA Nephropathy. | Xie et al., 2016 | 1 | 0 | 1 | 1 | 1 | 1 | 1 | 1 | 7 |
| Plasma exchanges for the treatment of severe systemic necrotizing vasculitides in clinical daily practice: Data from the French Vasculitis Study Group | Luna et al., 2015 | 1 | 1 | 1 | 1 | 1 | 1 | 1 | 1 | 8 |
| (POLARIS) A Prospective Observational Survey on the Long-Term Effect of LDL Apheresis on Drug-Resistant Nephrotic Syndrome | Muso et al., 2015 | 1 | 1 | 1 | 1 | 2 | 1 | 1 | 1 | 9 |
| Indications, technique, and outcome of therapeutic apharesis in European paediatric nephrology units | Paglialonga et al., 2015 | 1 | 1 | 1 | 1 | 1 | 1 | 1 | 1 | 8 |
| Rituximab in anti-GBM disease: A retrospective study of 8 patients | Touzot et al., 2015 | 1 | 0 | 1 | 1 | 0 | 1 | 1 | 0 | 5 |
| Long-Term Outcome of Anti-Glomerular Basement Membrane Antibody Disease Treated with Immunoadsorption | Biesenbach et al., 2014 | 1 | 0 | 1 | 1 | 0 | 1 | 1 | 0 | 5 |
| Effects of pretransplant plasmapheresis and rituximab on recurrence of FSGS in adult renal transplant recipients | Park et al., 2014 | 1 | 1 | 1 | 1 | 1 | 1 | 1 | 1 | 8 |
| Patients treated with therapeutic plasma exchange: A single centre experience | Samanci et al., 2014 | 1 | 1 | 1 | 1 | 2 | 1 | 1 | 1 | 9 |
| Success with plasmapheresis treatment for recurrent focal segmental glomerulosclerosis in paediatric renal transplant recipients | Straatmann et al., 2014 | 1 | 0 | 1 | 1 | 1 | 1 | 1 | 0 | 6 |
| Comparison of double filtration plasmapheresis with immunoadsorption therapy in patients with anti-glomerular basement membrane nephritis | Zhang et al., 2014 | 1 | 1 | 1 | 1 | 1 | 1 | 1 | 1 | 8 |
| Therapeutic plasmapheresis using membrane plasma separation. | Sinha et al., 2012 | 1 | 0 | 1 | 1 | 1 | 1 | 1 | 0 | 6 |
| ANCA-associated glomerulonephritis/systemic vasculitis in childhood: clinical features–outcome | Siomou et al., 2012 | 1 | 0 | 1 | 1 | 1 | 1 | 1 | 0 | 6 |
| Characteristics and outcome of crescentic glomerulonephritis in patients with both antineutrophil cytoplasmic antibody and anti-glomerular basement membrane antibody | Srivastava et al., 2012 | 1 | 0 | 1 | 1 | 1 | 1 | 1 | 0 | 6 |
| Anti-glomerular basement membrane disease: outcomes of different therapeutic regimens in a large single-centre Chinese cohort study | Cui et al., 2011 | 1 | 1 | 1 | 1 | 1 | 1 | 1 | 1 | 8 |
| Pre-emptive plasmapheresis and recurrence of focal segmental glomerulosclerosis in paediatric renal transplantation. | Gonzalez et al., 2011 | 1 | 1 | 1 | 1 | 1 | 1 | 1 | 1 | 8 |
| Plasmapheresis therapy in renal transplant patients: five-year experience | Gungor et al., 2011 | 1 | 1 | 1 | 1 | 1 | 1 | 1 | 1 | 8 |
| Long-term outcome of focal segmental glomerulosclerosis after paediatric renal transplantation. | Fuentes et al., 2010 | 1 | 1 | 1 | 1 | 1 | 1 | 1 | 1 | 8 |

**Supplemental Table 11:** Paediatric case reports and case series with different indications for plasmapheresis

| **Authors** | **Plasmapheresis type** | **Number of patients** | **Discussion** |
| --- | --- | --- | --- |
| **Lupus nephritis** | | | |
| Zhang et al[86] | Double filtration plasmapheresis | 5 | - Induction therapy was continued for six months with prednisone and additional immunosuppressive agents. - One passed away within two months of initiating DFPP secondary to lupus encephalopathy. - The cumulative removal rate of C3 was at 39.1% at the end of DFPP treatment. - There was a 4.55% incidence of having symptomatic hypotension. - There were two episodes of mild allergic reactions |
| **ANCA-associated vasculitis** | | | |
| Zhang et al | Double filter plasmapheresis | 3 | - One patient had crescentic glomerulonephritis with glomerulosclerosis. All patients in group II initially received steroid pulse therapy, followed by DFPP, prednisone, and cyclophosphamide and CNIs - After induction therapy for six months, one child remained on dialysis - In two dialysis-independent patients, one showed improvement in renal function and another required maintenance dialysis. - Improvements in extra-renal symptoms were noted. |
| Kouri et al | Plasmapheresis | 5 | - One patient who required plasmapheresis did not require concurrent KRT; 3 years following diagnosis, this patient remains off KRT CKD stage 3. - Of the remaining four patients who required KRT and were treated with plasmapheresis, two recovered renal function, both of whom were able to remain off dialysis at the most recent follow-up, with CKD stage 2 - One of the four patients was able to discontinue continuous venovenous haemofiltration after 25 days |
| Siomou et al[87] | Therapeutic plasma exchange | 10 | - Improvement of renal function was noted in five patients and stabilization in two patients - No improvement in three patients. - The renal function of the three patients who were not treated with plasma exchange remained stable. - Ten of 13 patients (77 %) achieved renal function improvement or stabilization. |
| **Anti-GBM disease** | | | |
| Zhang et al[86] | Double filter plasmapheresis | 1 | - Biopsy found crescentic glomerulopathy, RPGN. - The patient simultaneously received DFPP, prednisone, and cyclophosphamide for 4 sessions as an adjuvant induction therapy within two weeks. - After five months, the child achieved partial remission |
| **ABO-incompatible renal transplantation/hypersensitivity** | | | |
| Zhang et al[86] | Double filter plasmapheresis | 1 | - The patient received DFPP to achieve an anti-A and anti-B IgG titers lower than 1:16 pre- and post-operatively. - The anti-A and anti-B antibody titers fell progressively with each DFPP session, reaching less than 1:16 on the day of operation. - Post-operative anti-A IgM titers remained at 1:64 during the second week and rose to 1:128 one week later, necessitating another 3 DFPP sessions. - His anti-A IgM and IgG titers stabilized at 1:8 since four weeks after operation |
| Maxted et al[88] | Plasma exchange and immunoadsorption | 11 | - Eleven sensitized patients were treated using four different protocols of PE or IA, associated with RTX (in 5/11 children) and IVIG (6/11). - Two of them underwent both PE and IA. - Four patients were treated with IA (9–15 sessions) while they were on the rTx waiting list. - Three of them underwent a significant reduction in human leukocyte antigen (HLA) antibody titer. - The fourth child had a transient reduction in HLA antibody titer, but did not receive rTx. - Four patients were treated with PE alone while on the waiting list, without any reduction in antibody titer - A single patient with donor-specific antibodies underwent a living donor rTx with 3 pre- and two post-operative Pes with good post-transplant kidney function - The response of sensitized patients to PE/IA-based regimens was complete or partial in seven of the 11 cases (63.6 %). |
| **Focal segmental glomerulosclerosis** | | | |
| Shirai et al[89] | Plasmapheresis | 11 | - All 11 children included with primary FSGS and post-transplant recurrence showed significantly higher circulating levels of anti-nephrin autoantibodies. - Punctate IgG deposition co-localizing with nephrin was observed on graft biopsy. - After plasmapheresis, autoantibody levels reduced and patients attained complete remission with disappearance of IgG depositions on repeat graft biopsies. |
| Restrepo et al[90] | Plasma exchange | 17 | - Median number of plasma exchange procedures provided was 9 (IQR 6–11). - Complete remission was achieved by 15 patients after starting plasma exchange and augmenting immunosuppression - One patient died of a pulmonary thromboembolism within 2 weeks of transplant |
| Cleper et al[91] | Plasma exchange | 9 | - Plasma exchange used in nine of 14 recurrent primary FSGS transplants - Induced a complete and partial remission in eight of nine (only one patient did not respond to PP). - In the other patients, plasma exchange was not used due to the lack of availability - Although used for prolonged periods of time (up to three years during the study period), plasma exchange successfully preserved graft function |
| Paglialonga et al[92] | Plasma exchange | 13 | - Nine of the 13 patients treated with plasma exchange or immunoadsorption because of nephrotic syndrome had recurrent post-transplant FSGS. - Apheresis-based therapeutic regimens led to a partial or complete response in 9/9 patients with recurrent post-transplant FSGS (100 %). |
| Gonzalez et al[93] | Plasmapheresis | 17 | - Among the 15 LD patients, 13 received preemptive PP (1–10 sessions) and seven patients (47%) had subsequent FSGS recurrence. - Among the 19 DD patients, four received preemptive PP and 12 (63%) had FSGS recurrence. - The number of preemptive PP did not affect the recurrence rate. - In a group of patients with a previous graft loss secondary to recurrence, the rate of recurrence was lower than expected (40%) and two of the three patients who did not recur had three or more sessions of preemptive PP. - Of the 19 patients with recurrence, 17 were treated with PP therapy and 88% of the patients fully or partially responded. Only five patients had graft loss at three years post-transplant: two from FSGS recurrence and three from non-compliance. - These results suggest that preemptive PP does not decrease the rate of recurrence after transplantation but might be beneficial in treating high-risk patients with documented recurrence. |
| Fuentes et al[94] | Plasmapheresis | 7 | - Plasmapheresis was performed in seven of nine patients who had suffered recurrence in the first graft. - The number of sessions varied between six and 12. - Two patients underwent plasmapheresis immediately after transplantation. - Neither of these patients experienced recurrence of FSGS. - The clinical response (proteinuria <0.5 g/day) was dominant in the group undergoing plasmapheresis (85.7% vs 0%, P = 0.083), although this did not reach statistical significance, likely due to the low number of patients. |
| Artero et al[95] | Plasmapheresis | 9 | - Obtained complete remission in six of nine children and adults. |
| Andresdottir et al[96] | Plasma exchange | 7 | - Achieved complete remission in three patients and partial remission in two. |
| Greenstein et al[97] | Plasmapheresis | 6 | - Recurrent FSGS in 8/20 paediatric renal transplants. - Performed plasmapheresis in six of these eight patients and achieved remission in five (initiation of therapy was delayed in the patient who did not respond to PP). |
| Dall’Amico et al[98] | Plasmapheresis | 11 | - Observed in 15 out of 29 children after the first transplantation. - Used plasmapheresis and cyclophosphamide in 11 of the 15 patients with recurrence and obtained persistent remission in seven of them (63.6%). |
| Ohta et al[99] | Plasmapheresis | 5 | - Observed recurrence of FSGS in 9/21 paediatric renal transplants. - The incidence of recurrence was lower in patients who had undergone pre-operative PP (5/15) than in those patients who had not (4/6). |

| **Study** | **LDL-A method** | **Patients** | **Results** |
| --- | --- | --- | --- |
| Muso et al | Dextran sulfate | 17 | - LDL-A used for 12 sessions, with full-dose steroids and lipid-lowering agents - Serum cholesterol and phospholipid levels were significantly reduced only in the LDL-A group compared to steroid monotherapy - LDL-A significantly decreased urinary protein and increased serum albumin - The average time to reduce urine protein to below the nephrotic range (< 3.5 g/day) was significantly shorter using LDL-A |
| Stevinkel et al | Dextran sulfate | 7 | - LDL–A used 2 times per week for 3 weeks and then once a week for 7 weeks. - Remission of nephrotic syndrome in 2 patients, clear improvement in 4 patients - Significantly decreased serum cholesterol, apoB, and plasma lipoprotein A levels |
| Brunton et al[100] | Dextran sulfate | 10 | - LDL-A used 12 times over a period of 6 to 12 weeks - Total cholesterol significantly lowered - Two apheresed patients had a prolonged remission |
| Kitajima et al[101] | Dextran sulfate | 4 | - LDL-A group had higher urinary protein excretion (13.7 vs. 5.2 g/day, P = 0.053) and serum creatinine (4.11 vs. 1.65 mg/dL) levels at onset, and a higher remission rate (75.0% vs. 58.7%) - 1 patient failed to respond to treatment and developed kidney failure |
| Shah et al[75] | Liposorber LA-15 | 7 | - All the patients in this series after undergoing LDL-A experienced at least ten-fold reductions in proteinuria, achieving and sustaining levels of proteinuria less than the nephrotic range - 4 patients experienced complete remission of post-transplant FSGS |
| Sannomiya et al[70] | Dextran sulfate | 5 | - No signs or symptoms suggesting recurrence of FSGS in any of the patients. |

| **Haemolytic uremic syndrome** | | | |
| --- | --- | --- | --- |
| Paglialonga et al[92] | Plasma exchange and double filter plasmapheresis | 12 | - Two children with typical post-diarrheal HUS had complete remission - One child with post-pneumococcal HUS partially responded. - One child had TTP and achieved a complete response after five plasma exchanges. - In seven of these cases, plasma was used as the substitution fluid (1–27 sessions, followed by eculizumab in two cases). - A complete and a partial response were observed among two children with factor H auto-antibodies treated with 16 and 17 sessions, respectively; no effect was obtained after six sessions in a patient with membrane cofactor protein (MCP) deficit. - Two of the remaining four patients (all without defined genetic mutations) completely responded, and two experienced a partial response. - One patient with HUS associated with factor H auto-antibodies had been chronically treated with PE (1.5 times plasma volume per session) every 3–4 weeks for 9 years but eventually achieved stable remission. |
| Khandelwal et al[59] | Plasma exchange | 109 | - Haematological remission (defined as platelet count of >100 000/μL, schistocytes <2% and LDH <450 U/L) was achieved in 73 (98.6%) and 32 (91.4%) patients with and without anti-FH associated disease - 65% of patients achieved remission within 1 week of initiation plasma exchange - One month after starting PEX, 19.2% of patients still required dialysis, but by three months, 89.6% were dialysis-free - At a median follow-up of 17.5 months, over half had normal renal function with hypertension and/or proteinuria, while 15.1% had CKD stage 2-3. |
| Johnson et al[60] | Plasmapheresis | 71 | - Median time to enter haematological remission was 11.5 days - 8 of 71 (11 %) patients did not enter haematological remission by day 33. - 12 patients (17 %) required dialysis at day 33. |

**Supplemental Table 12:** Comprehensive protocol for lipoprotein apheresis

| **Indications:**   - Refractory nephrotic syndrome (e.g., FSGS, drug-resistant nephrotic syndrome), hyperlipidemia unresponsive to pharmacotherapy, or other lipid-related pathologies (e.g., familial hypercholesterolemia).   **Pre-Treatment Evaluation:**   - **Patient selection:** Assess patient’s renal condition, lipid profile, and overall health status. - **Contraindications:** Rule out any contraindications such as ACE inhibitor use, severe coagulopathy, or active infections that may complicate apheresis. - **Pre-procedure labs:**   - Lipid profile   - Renal function tests (serum creatinine, GFR).   - Coagulation profile and complete blood count.   **Procedure Setup:**   - **Technique selection:** Use either dextran sulfate cellulose adsorption, HELP (heparin-induced extracorporeal LDL precipitation) system, DALI, or a filter with antibodies to lipoprotein.   - Double filtration plasmapheresis: Plasma is initially separated using centrifugation or a primary membrane separator. It then passes through a secondary membrane in LDL-apheresis, which has a cutoff around one million daltons. Molecules larger than this, such as LDL cholesterol (~2.3 million daltons), are retained, while smaller components pass through and are returned to the patient.   - Immunoadsorption: Plasma is passed through antibody coated columns which are flushed with saline and regenerated by a glycine buffer.   - HELP: Plasma is mixed with an acid buffer, which is added to heparin. LDL cholesterol is precipitated and then filtered out through this process, although it is non-selective.   - DALI: Blood is passed through a polyacrylate-coated polyacrylamide adsorber, which interacts with LDL.   - Dextran sulfate: Low molecular dextran sulfate is used to selectively bind LDL. - **Vascular access:** Establish adequate vascular access, typically through a central venous catheter or AV fistula for high blood flow. - **Replacement fluid:** In most cases, replacement fluids such as albumin or saline are not needed due to the selective removal of LDL particles. - **Anticoagulation:** Administer anticoagulation to prevent clotting in the extracorporeal circuit, typically with low doses of heparin or citrate. - **Need for blood priming:** The plasma circuit (170 mL) should be blood-primed for patients under 25 kg or with hematocrit below 25%   **Treatment Protocol:**   - **Session duration:** Each LDL-apheresis session lasts about 2–3 hours. - **Frequency:** Typically performed once a week initially, and adjusted based on clinical response (e.g., every 1–3 weeks). - **Blood flow rate:** Can range 50-120 mL/min; typically use 2X weight (for a 40 kg patient, use 80 mL/min) - **Plasma flow rate:** Should be 20-30% of blood flow rate - **Typical exchange volumes:** LDL-A: Varies depending on the lipid load and clinical indication, typically 2-3 liters of plasma are processed. - **Post-apheresis access instructions:** Lock catheter with TPA/Sodium citrate - **Number of sessions:** Usually between 6 and 12 sessions, depending on the patient’s response and disease progression. - **Monitoring during procedure:** Continuous monitoring of blood pressure, heart rate, and symptoms. Regular checks of ionized calcium and electrolytes (especially in patients on citrate anticoagulation).   **Post-Treatment Care:**   - **Monitoring:** Regular follow-up to assess kidney function (serum creatinine, GFR), proteinuria, and lipid levels. Track improvements in nephrotic syndrome symptoms (e.g., edema, proteinuria). - **Adjunctive therapy:** Continue or adjust immunosuppressive therapy or steroids, if applicable, to enhance the overall treatment outcome. - **Patient education:** Provide information on signs of adverse events, such as hypocalcemia or allergic reactions, and ensure prompt reporting of any complications.   **Adverse Event Management:**   - **Hypocalcemia:** Monitor calcium levels during and after the procedure. Administer calcium supplements if needed. - **Allergic reactions:** Pre-medicate with antihistamines or corticosteroids if needed, particularly if using fresh frozen plasma for replacement. - **Hypotension or vascular complications:** Address by adjusting the blood flow rate, providing saline infusion, or modifying anticoagulation.   **Long-Term Monitoring:**   - **Renal function:** Monitor kidney function for long-term improvements or stability post-apheresis. - **Lipid profile:** Regular lipid panel checks to evaluate the effectiveness of LDL reduction. - **Disease recurrence:** In conditions like FSGS, monitor for recurrence of proteinuria and adjust therapy as needed.   **Documentation and Follow-Up:**   - **Record each session:** Document the duration, parameters, and any complications during the apheresis session. - **Response to therapy:** Track clinical improvements, reduction in proteinuria, and stabilization of kidney function over time.   **Special Considerations:**   - **Elderly patients:** Consider coexisting conditions, particularly cardiovascular risks, when determining suitability for LDL-apheresis. |
| --- |

**Supplemental Table 14:** GRADE Summary of Pooled Clinical Outcomes for Patients Undergoing Apheresis for Glomerular Diseases

Patient or Population: Children and adults with glomerular diseases
Settings: Hospital/inpatient and outpatient
Intervention: Therapeutic apheresis
Comparison: (Standard care - No apheresis)

| **Outcomes** | **Anticipated comparative risks* (95% CI)** | **No. of participants (studies)** | **Quality of the evidence (GRADE)** | **Comments** |
| --- | --- | --- | --- | --- |
| Remission | 607 per 1000 (474–732) | 420 (17 studies) | **C** | Substantial heterogeneity (I² = 85.8%); variable definitions and indications; mostly retrospective data. |
| Kidney Failure | 264 per 1000 (174–365) | 1,005 (18 studies) | **C** | High risk of bias; high heterogeneity (I² = 90%); outcomes pooled across diseases. |
| Dialysis requirement | 304 per 1000 (148–488) | 356 (14 studies) | **C** | Very wide CIs; imprecise effect; marked heterogeneity (I² = 91%). |
| Mortality | 109 per 1000 (91–128) | 1,114 (27 studies) | **B** | Lower heterogeneity (I² = 48%); still predominantly observational data. |
| Adverse events | 310 per 1000 (128–531) | 344 (9 studies) | **C** | High risk of bias; Higher heterogeneity(I² = 93%); very wide range; underreporting possible. |

** The basis for the anticipated risk is the event rate pooled proportion across included studies, with 95% confidence intervals. Most studies compare outcomes before and after apheresis, or are uncontrolled observational cohorts.*

**CI:** Confidence interval

**GRADE Working Group grades of evidence:**High quality (A): Further research very unlikely to change confidence in estimate.
Moderate quality (B): Further research likely to have an important impact and may change the estimate.
Low quality (C): Further research very likely to have an important impact and is likely to change the estimate.
Very low quality (D): We are very uncertain about the estimate.

**Explanations:**

- All outcomes start at “low” due to observational design.
- Most outcomes were downgraded for risk of bias, heterogeneity, and imprecision.
- Mortality rated “moderate” due to more consistent reporting.
